# The dominant *Anopheles *vectors of human malaria in the Americas: occurrence data, distribution maps and bionomic précis

**DOI:** 10.1186/1756-3305-3-72

**Published:** 2010-08-16

**Authors:** Marianne E Sinka, Yasmin Rubio-Palis, Sylvie Manguin, Anand P Patil, Will H Temperley, Peter W Gething, Thomas Van Boeckel, Caroline W Kabaria, Ralph E Harbach, Simon I Hay

**Affiliations:** 1 Spatial Ecology and Epidemiology Group, Tinbergen Building, Department of Zoology, University of Oxford, South Parks Road, Oxford OX1 3PS, UK; 2 BIOMED, Universidad de Carabobo, Apartado 2073, Maracay 2101-A, Venezuela; 3 Laboratorio de Ecología de Vectores, Dirección de Control de Vectores y Fauna Nociva, Ministerio del Poder Popular para la Salud, Maracay, Venezuela; 4 Institut de Recherche pour le Développement, Lab. d'Immuno-Physiopathologie Virale et Moleculaire, UMR-MD3/Univ. Montpellier I, Faculté de Pharmacie, 15, Ave Charles Flahault, 34093 Montpellier, France; 5 Biological Control and Spatial Ecology, Université Libre de Bruxelles CP160/12, Av FD Roosevelt 50, B1050, Brussels, Belgium; 6 Malaria Public Health and Epidemiology Group, Centre for Geographic Medicine, KEMRI - Univ. Oxford - Wellcome Trust Collaborative Programme, Kenyatta National Hospital Grounds, P.O. Box 43640-00100 Nairobi, Kenya; 7 Department of Entomology, The Natural History Museum, Cromwell Road, London, UK

## Abstract

**Background:**

An increasing knowledge of the global risk of malaria shows that the nations of the Americas have the lowest levels of *Plasmodium falciparum *and *P. vivax *endemicity worldwide, sustained, in part, by substantive integrated vector control. To help maintain and better target these efforts, knowledge of the contemporary distribution of each of the dominant vector species (DVS) of human malaria is needed, alongside a comprehensive understanding of the ecology and behaviour of each species.

**Results:**

A database of contemporary occurrence data for 41 of the DVS of human malaria was compiled from intensive searches of the formal and informal literature. The results for the nine DVS of the Americas are described in detail here. Nearly 6000 occurrence records were gathered from 25 countries in the region and were complemented by a synthesis of published expert opinion range maps, refined further by a technical advisory group of medical entomologists. A suite of environmental and climate variables of suspected relevance to anopheline ecology were also compiled from open access sources. These three sets of data were then combined to produce predictive species range maps using the Boosted Regression Tree method. The predicted geographic extent for each of the following species (or species complex*) are provided: *Anopheles *(*Nyssorhynchus*) *albimanus *Wiedemann, 1820, *An*. (*Nys*.) albitarsis*, *An*. (*Nys*.) *aquasalis *Curry, 1932, *An*. (*Nys*.) *darlingi *Root, 1926, *An*. (*Anopheles*) *freeborni *Aitken, 1939, *An*. (*Nys*.) *marajoara *Galvão & Damasceno, 1942, *An*. (*Nys*.) nuneztovari*, *An*. (*Ano*.) pseudopunctipennis* and *An*. (*Ano*.) *quadrimaculatus *Say, 1824. A bionomics review summarising ecology and behaviour relevant to the control of each of these species was also compiled.

**Conclusions:**

The distribution maps and bionomics review should both be considered as a starting point in an ongoing process of (i) describing the distributions of these DVS (since the opportunistic sample of occurrence data assembled can be substantially improved) and (ii) documenting their contemporary bionomics (since intervention and control pressures can act to modify behavioural traits). This is the first in a series of three articles describing the distribution of the 41 global DVS worldwide. The remaining two publications will describe those vectors found in (i) Africa, Europe and the Middle East and (ii) in Asia. All geographic distribution maps are being made available in the public domain according to the open access principles of the Malaria Atlas Project.

## Background

There is increasing knowledge of the global risk and distribution of *Plasmodium falciparum *malaria [1] and the intensity of its transmission [2], which reveals the nations of the Americas to have the lowest *P. falciparum *malaria endemicity worldwide [2] and hence the lowest *P. falciparum *morbidity [3,4]. Work is ongoing to develop the same cartographic suite for *P. vivax *[5]. Data from national health information system reporting support these findings and additionally shows a near cosmopolitan decrease in *P. falciparum *and *P. vivax *malaria morbidity and mortality across the continents between 2000 and 2007 [6].

Key to these gains, has been the sustained integrated vector control strategy [7-12] championed in the "*Regional strategic plan for malaria in the Americas 2006-2010*" [13]. Moreover, these successes and a transformed funding environment [14] have encouraged three of the 21 malaria endemic nations in the Americas (Argentina, El Salvador and Mexico), to scale-up their control ambitions [13] and explicitly target the elimination of malaria within their territories [15]. The global strategic framework for integrated vector management (IVM) calls for "*an evidence-based decision-making approach which involves the adaptation of strategies and interventions to local vector ecology, epidemiology and resources that are guided by operational research and subject to routine monitoring and evaluation*" [16]. Integrated vector control will therefore remain central to sustaining control impact and become increasingly important for those nations targeting the higher ambition of elimination [17].

Recent attempts to map *Anopheles *distributions have been made in the Americas [7,18-23]. These include an assessment of mosquito species richness and endemicity in the Neotropical Region (data based on the Mosquito Information Management Project (MIMP) database, incorporating museum specimens dating from 1899 to 1982) [18]; expert opinion ranges for five American DVS, at a continental scale [23]; point data maps detailing the distribution of major malaria vectors in Venezuela from the early 1900s to 2005 [21]; the predicted distribution of members of the *An. quadrimaculatus *Subgroup [19]; the mapped results of a 35-year anopheline survey effort across Panama [20]; and an eco-regional map with details of each region's associated vectors [22]. The evidence-base of occurrence records is not always provided and the mapping techniques used range from those based on expert opinion and simple interpolations to those employing more sophisticated statistical methods. Consequently, the resulting maps are difficult to compare and impossible to synthesize at the continental scale. In addition, species sympatry often obscures the relative contribution to local human malaria transmission. These interactions can, in part, be clarified by an overview of the life history characteristics (bionomics) of vector species pertinent to epidemiology and control.

The current paper focuses on the work carried out on the nine dominant vector species (DVS) identified in the Americas [24] (Table 1). Occurrence data assemblies, expert opinion map compilation and range predictions for each species (or species complex) are presented, alongside a summary of the behaviour and bionomics relevant to local malaria epidemiology and intervention. The DVS of other geographical regions [24] including Africa, Europe and the Middle-East (Sinka *et al*: The dominant *Anopheles *vectors of human malaria in Africa, Europe and the Middle East: occurrence data, distribution maps and bionomic précis, unpublished) and Asia (Sinka *et al*: The dominant *Anopheles *vectors of human malaria in the Asia Pacific region: occurrence data, distribution maps and bionomic précis, unpublished) will be considered in future publications.

**Table 1:** Defining the dominant *Anopheles *vector species and species complexes of human malaria in the Americas.

Anopheline species or species complex	**White **[29]	**Service **[27,28]	**Kiszewski **[25]	**Mouchet **[26]	**Exc**.	**Inc**.	TAG final	EO source
*albimanus *Wiedemann, 1820: *An*. (*Nyssorhynchus*)	1, 2, 3	1, 2, 3	1, 2, 3	1, 2, 3	1	1	1	Rubio-Palis (unpub. obs., 2008), Manguin (unpub. obs., 2009), Fernandez (unpub. obs., 2009), Updated by TAG (2009).
albitarsis*: *An*. (*Nyssorhynchus*)	3	3				1	1	[94], [200], Rubio-Palis (unpub. obs., 2009), Manguin (unpub. obs., 2009), Updated by TAG (2009).
*aquasalis *Curry, 1932: *An*. (*Nyssorhynchus*)	2, 3	2, 3	2, 3	2, 3	1	1	1	[26], Rubio-Palis (unpub. obs., 2008).
*darlingi *Root, 1926: *An*. (*Nyssorhynchus*)	2, 3	2, 3	2, 3	2, 3	1	1	1	[26], Rubio-Palis (unpub. obs., 2008), Updated by TAG ( 2009).
*freeborni *Aitken, 1939: *An*. (*Anopheles*)	1	1	1			1	1	[201]
*marajoara *Galvão & Damasceno, 1942: *An*. (*Nyssorhynchus*)			2, 3			1	1	[200], [202], Rubio-Palis (unpub. obs., 2009), Manguin (unpub. obs., 2009).
nuneztovari*: *An*. (*Nyssorhynchus*)	3	3	3	3	1	1	1	[26], Rubio-Palis (unpub. obs., 2009), Updated by TAG (2009).
pseudopunctipennis*: *An*. (*Anopheles*)	1, 2, 3	1, 2, 3	1, 2, 3	1, 2, 3	1	1	1	[26,148], Rubio-Palis (unpub. obs., 2009), Updated by TAG (2009).
*quadrimaculatus *Say, 1824: *An*. (*Anopheles*)	1	1	1	1	1	1	1	[201]

* denotes that a "species" is now recognized as a species complex. The exclusive (Exc.) column counts those species identified in all four listed reviews. The inclusive (Inc.) column counts those species identified by any of the four reviews and are therefore the candidate dominant vector species (DVS) considered for mapping. The numbers given in each of the review author columns record the Macdonald malaria epidemiology zone [199] in which the species can be found: 1 - North American; 2 - Central American; 3 - South American. The final DVS species listed were defined during two meetings of a Technical Advisory Group (TAG). EO = Expert opinion.

## Methods

### Species selection

The selection of DVS has been previously described [24]. Briefly, a series of authoritative review articles were consulted [25-29], and those *Anopheles *vectors identified as main, dominant or principal were assembled to give an inclusive list of 52 species globally. This list was further refined in consultation with a Technical Advisory Group (TAG) of vector experts to give a final list of 41 species and species complexes worldwide, nine of which are found in the Americas (Table 1) [24].

### Data assembly - maps

The Malaria Atlas Project (MAP) parasite rate data library was searched first [30], augmented with a systematic search, conducted in January 2007, for published, peer-reviewed literature detailing primary anopheline vector occurrence data. Online scientific bibliographic databases (PubMed [31] and Web of Science [32]) were searched for post 31 December 1984 articles using "*Anopheles*" as the keyword search term. In addition, relevant electronic archives were also searched, including AnoBase [33], the Walter Reed Biosystematics Unit (WRBU) Mosquito Catalog [34], the Disease Vectors Database [35], archives of MalariaWorld [36] and Malaria in the News [37], and a review of selected bibliographies [26].

The resulting citation library was then reviewed and refined, retaining all references that met the following criteria for inclusion: (i) the reported study was undertaken after December 1984 (longitudinal surveys that began prior to but continued past this date were included); (ii) the surveys provided location information to a precision of administrative unit level one or higher; (iii) the surveys reported primary data; (iv) the surveys provided species-level information at the studied location; and (v) the surveys reported the presence of at least one DVS. Electronic mail alerts were then set up on various malaria information bulletins (Malaria in the News [37], Malaria World [36] and the Malaria Bulletin [38]) and by using "*Anopheles*" as a key word to generate article alerts *via *PubMed [31]. Content notifications for specific high yielding journals including *Malaria Journal *and *Parasite and Vectors *(*via *BioMed Central [39]), *Vector-Borne and Zoonotic Diseases *[40] and the *Indian Journal of Medical Research *[41] were also set up. Results from these searches were included until 31 October 2009.

Globally, the literature search resulted in 3857 publications or reports containing potential data to be reviewed. Of these publications, 2276 fulfilled the inclusion criteria, providing data for 147 countries worldwide. A total of 366 sources detailed surveys conducted across 25 countries in the Americas that are further summarized in the results. These data were then augmented with species records from the WRBU MosquitoMap [42].

### Expert opinion distribution maps

Expert opinion (EO) maps were digitised from exhaustive searches of published distribution maps (Table 1). These were then refined by the TAG of *Anopheles *experts (see acknowledgements) during a meeting held in Oxford (23-25 September 2009). A compressed file containing these EO distributions in ArcGIS format is provided as Additional file 1: Expert opinion distribution maps for the nine DVS of the Americas.

### Database

The data were extracted into Microsoft Excel datasheets using a protocol provided elsewhere [24]. In brief, from each source article, the first author, date of publication, publication type (published article, thesis, unpublished report, *etc*.) were recorded. Vector- and survey-specific data abstracted included: survey location(s) including country- and administration-level information where given; whether the survey was conducted in an urban or rural location (as defined in the data source); the presence of forest or rice growing areas within the survey area (as defined in the data source); month, year and duration of the survey including information relating to multiple or longitudinal surveys where reported; species data, including sibling information where given; all vector sampling techniques employed in the survey (*e.g*. human landing indoors and outdoors, animal bait, resting indoors and outdoors); all identification methods (*e.g*. morphology, polymerase chain reaction (PCR) methods, cross-mating); and any control programs or methods currently in place in the survey location(s) (*e.g*. insecticide treated bednets (ITNs), insecticide residual spraying (IRS) or larvicide applications). No assumptions were made in the data abstraction, with all reported data accurately reflecting the level of detail given in the data source. For example, where a methodology described a series of monthly surveys conducted over a year, but the results were amalgamated and presented for the whole survey period, these data were abstracted reflecting the whole survey period. Monthly data were only recorded where survey results specifically reported the presence of the vector species in question during that month's survey.

The data then underwent a three-level checking procedure, with the first level check conducted by a different abstractor to ensure an independent assessment of the assembled data. All aspects of the data were reviewed to ensure the information had been correctly collated and the sites geo-referenced accurately. Detailed notes were included to describe how each line of data had been checked and where changes had been made. The second-level check (by MES) incorporated suggested changes with an emphasis on geo-referencing and the examining of evidence given in support of the choice of coordinates.

Once these first- and second-level checks were completed, the Excel sheets were migrated into a web-based PostgreSQL database [43] with a custom-designed Python interface (based on Django [44]). The Python interface is important because it allows automated production of the occurrence data maps for review, and communication between the database and the PyMC statistical language ultimately used to produce the predicted ranges.

Third-level checks were implemented using the database to identify: (i) inconsistent or non-standard spellings within fields that would affect query summaries; (ii) blanks in mandatory data fields; (iii) any occurrence data that fell in the sea or other major water body (those falling in water bodies less than 1 × 1 km from the land were automatically adjusted, any points falling a distance greater than 1 × 1 km from the land were manually checked and corrected); (iv) any suspect geo-locations that fell outside the allocated country boundary; (v) all multi-point locations within a point or wide-area area type (*e.g*. where a survey listed three geo-referenced villages, but the data were reported across the whole survey area) in order to identify the point most central to the entire survey area.

Preliminary maps were produced, displaying the EO ranges and the occurrence data. Any points that fell outside the EO outline were checked and accepted as valid, or rejected, based on information provided by the TAG. The final occurrence data and EO maps were collated to show the extent of the geographical distribution of each species and to allow gaps in the data retrieved to be identified [24]. These are shown as thumbnails in the predictive maps in Additional file 2: Predictive species distribution maps for the nine DVS of the Americas.

### Boosted Regression Tree modelling

A wide range of approaches have been developed for empirical modelling of species distributions given data on point observations of occurrence. In this study, the Boosted Regression Tree (BRT) method [45,46] was chosen to generate predictive maps of *Anopheles *species distribution. This selection was based on a number of factors: first, in a review of 16 species modelling methods, BRT was one of the top performing methods evaluated using the Area Under the receiver operating characteristic Curve (AUC) and correlation statistics [47]; second, the method is flexible in being able to accommodate different types of predictor variables (*e.g*. continuous or categorical data); third, it is easy to understand and uses reliable, well documented and freely available R code; and fourth, the resulting maps are simple to interpret and include a ranked list of environmental predictors.

A full description of the BRT method is given in Elith *et al*. [45]. Briefly, BRTs combine regression or decision trees and "boosting". Regression trees use binary recursive partitioning to iteratively split the data into partitions. Put simply, the model uses the data (in this case presence and pseudo-absence (see below) of a mosquito species) and, in a series of steps, identifies the threshold of each input variable that results in either the presence or the absence of the species. It allows the input of continuous and categorical variables and different scales of measurement amongst the predicting variables.

Boosting is a machine-learning algorithm that increases a model's accuracy [45,46,48]. This is applied to the regression trees to help improve their predictive performance. Boosting is based on the principle that in machine learning, a set of "weak" rules can create a single "strong" or accurate rule, and it is easier to create many of these weak rules than it is to find a single accurate rule. For example, it is easy to generalise and state that, as a rule of thumb, a certain anopheline species is never found above a certain altitude, however, accurately specifying the exceptions to this rule across the species' range is considerably more difficult. In the case of BRT, a set of regression trees, each describing the data to a greater or lesser extent, are combined, or boosted, to create a single model with a higher predictive performance than each of its composing parts.

The boosting process within the BRT attempts to minimise a loss function, in this case deviance (two times the log of the ratio of the likelihood of the data, given the fitted model, and the likelihood of the data, given a perfectly fitting model) that indicates the poor performance of a weak model. Starting with the regression tree within the set that explains the most deviance, additional trees are added that best fit the residuals, or unexplained deviance, of the first tree. The residuals of this new two-tree model are recalculated and the next tree is added to fit these updated residuals, and so on. This stage-wise method could theoretically continue until the model is completely over-fitted, and predictive performance lost. To prevent such over-fitting, shrinkage (reducing the contribution of each tree) is applied to each new tree as it is added which involves the optimisation of the number of trees within the final model, the learning rate of the boosting (which is inversely related to the number of trees in the model), and the final tree complexity. This optimisation is achieved using cross-validation, where the model is tested using a sub-sample of withheld data, before the final model is run using all the data, applying the predetermined optimal settings.

### Evaluation statistics

Deviance (see above), Correlation, Discrimination (AUC) and Kappa (κ) summary statistics were calculated to accompany each map combining classic accuracy metrics (*e.g*. Kappa) and those more specific to, and calculated by, the BRT method and recommended for use in Elith *et al*. [45] (*e.g*. Deviance). These statistics are used here as a guide to the predictive performance of the maps, but not to compare one map to another, as they were each generated using different datasets.

During cross-validation, BRT repeatedly partitions the data into training and testing subsets and performs cross-validation on each of the different partitions (or folds). It then calculates the mean and standard error for each statistic. The deviance metric is the prime measure of accuracy for the BRT method, and indicates the proportion of deviance still unexplained by the model. The lower the deviance value (minimum value = 0), the better the model is at predicting the hold-out sets. Correlation simply correlates the predicted probability of presence against the occurrence data, giving a value between -1 and 1. The area under the curve (AUC) of the receiver-operator characteristic is also presented as an accuracy metric. The AUC provides values ranging between 0 and 1, where 1 is a perfect prediction, and is an index of the area under the curve of a plot of sensitivity (the proportion of the testing set that is correctly classified as a presence) against specificity (the proportion that is correctly classified as an absence) as the classification threshold is increased [49]. Kappa is an index of the proportion of agreement of predicted versus observed positive and negative samples, calculated from an error matrix that cross references the number of observed and the number of predicted pixels categorised as presence or absence [49-51]. It can provide values ranging from -1 to 1, where values less than zero indicate that the model is worse than random, zero shows that the model is no better than random, and greater than zero indicates that the model performs better than random.

### Environmental and climatic variables

Each of the environmental or climatic data grids described below have undergone a number of processing steps prior to being used in the BRT. Each grid was post-processed to ensure that the pattern of land and sea pixels corresponded exactly across all grids, in order to provide a suite of spatially identical grids. This required trimming or gap-filling *via *nearest-neighbour interpolation to reconcile minor misalignments in coastline definitions or, for example, small data gaps due to cloud error.

Covariates were chosen based on factors known to influence anopheline ecology and therefore include elevation [52], climatology surfaces interpolated from networks of meteorological stations [53], and remotely sensed data from Earth observation satellites in their raw form and categorised into global land cover maps [54,55]. Where remotely sensed imagery was available as multi-temporal data, temporal Fourier analysis (TFA) was used to ordinate the data by decomposing the temporal signal into an additive series of harmonics of different seasonal frequencies [56]. The TFA algorithm [56] generated seven products for each temporal variable: the overall mean, maximum and minimum of the data cycles; the amplitude (maximum variation of the cycle around the mean) and the phase (the timing of the cycle) of the annual and bi-annual cycles. The grids are all described in detail below.

### Elevation

The Shuttle Radar Topography Mission (SRTM), consisting of a synthetic aperture radar on board the Space Shuttle Endeavour, flew an 11 day mission to create a near-global high spatial resolution digital elevation model (DEM) [52,57]. The DEM grids used here are processed by applying land/sea masks and the data are projected into the MODIS Land (MODLAND) tile system. The MODerate Resolution Imaging Spectroradiometer (MODIS) is a NASA satellite sensor which provides 1 × 1 km spatial resolution satellite imagery globally [58].

### Worldclim database

The Worldclim database consists of a freely available set of global climate data at a 1 × 1 km spatial resolution compiled using weather data collected from world-wide weather stations [53]. The data spans from 1950-2000 and describes monthly precipitation and mean, minimum and maximum temperatures. From these data, interpolated climate surfaces have been produced using ANUSPLIN-SPLINA software [59].

### Advanced Very High Resolution Radiometer

The Advanced Very High Resolution Radiometer (AVHRR) 8 × 8 km products are available over a 20-year time series, and a limited series of 1 × 1 km resolution data are only available for April to December 1992; January to September 1993; February to December 1995 and January to April 1996. Both data series were downloaded (Goddard Space Flight Center's Distributed Active Archive Center on the Global Land Biosphere Data and Information Web Site [54]) and processed for use here [60].

The AVHRR grids used include the normalized difference vegetation index (NDVI), land surface temperature (LST) and middle infrared radiation (MIR). The NDVI numerically indicates the level of green, photosynthesizing, and therefore active, vegetation derived from the spectral reflectance of AVHRR channels 1 and 2 (visible red and near infrared wavelength, respectively) [61,62]. The LST index uses thermal infrared radiometry to measure land temperature, corrected for atmospheric influences, such as water vapour, aerosols, carbon dioxide or ozone [61,63]. Finally, the MIR data is applied to discriminating land cover. It is able to penetrate more fully than shorter wavelengths through aerosol particles, including atmospheric water, and it is considered better able to distinguish between vegetation, soil, rock and water [61,64].

### Globcover project

The Globcover project [55] provides satellite-derived land cover maps from the MERIS spectrometer on board ENVISAT. The data produced are at a 300 × 300 m resolution and the satellite imagery goes through a number of pre-processing and classification steps prior to map production, which include cloud screening and shadow detection, water reclassification and atmosphere (including aerosol) correction. The final map products include global land cover mosaics for the period from December 2004 to June 2006, providing 22 land cover classes; and regional mosaics, which detail up to 51 land cover classes. Due to the limited areas covered by the regional mosaics, the global mosaics are used here. They include land-cover classes particularly relevant to mosquito habitats, for example, post-flooding or irrigated croplands, rain-fed croplands, urban areas and numerous categories for forests, including those regularly flooded with fresh or saline/brackish water. The 22 categories were available to the BRT individually and also grouped into three land cover types: flooded areas, forested areas and dry areas. Finally, to produce grids of an equivalent resolution to the other environmental and climatic variables applied in the vector mapping (5 × 5 km), the 300 m mosaics were re-sampled using a majority filter where the most common class in each pixel subset was identified and used to redefine the new, larger pixel.

The covariate sources detailed above provide a range of spatial, high resolution environmental or climatic open source data freely available for species mapping. All grids were available to the BRT mapping process allowing the model to identify which variable, or suite of variables most accurately described the species distribution.

### Mapping protocol

Numerous model iterations were run with varying combinations of buffer size, number of pseudo-absences and source data (hybrid data: occurrence data plus pseudo-presences randomly assigned from within the EO species range; EO data: pseudo-presences randomly assigned from within the EO range; and occurrence data only). As each of these categories required the use of different data inputs to the BRT, statistical comparison using the evaluation metrics was not possible. Therefore the "optimal" settings chosen are subjective and based on visual examination and comparison of the various maps guided by, but not relying on, the evaluation statistics.

The lack of true absences in the occurrence data is a common problem encountered with species mapping and can be addressed by the production of pseudo-absences. These can be created in a number of ways [65] including creating background samples within the region of study [47,66] to represent the set of conditions available to the species, though not necessarily areas where the species is genuinely absent. Alternatively, and to minimise the risk of including unidentified presence data, pseudo-absences can be assigned within a buffer zone surrounding the region of study [49,67]. This second method is particularly useful where the extent of the species range to be mapped is known or can be reasonably estimated, for example with expert opinion maps. The buffer in such cases represents an area that is not geographically implausible for the species to reach, but maybe outside the limit of the environment that the species can tolerate. The creation of pseudo-absences in the buffer zone was used here.

The size of the buffer zone can have implications for the accuracy and viability of the final mapping outputs [67]. A buffer that is too large, allowing pseudo-absences to be generated far from the presence data, may result in a model that is defining regional or coarse geographical differences rather than the fine-scale variables that influence the presence or absence of a species. Conversely, a buffer that is too small, which relies on pseudo-absences generated too close to the presence data, risks incorporating unknown areas of presence, severely limiting the ability of the model to distinguish presence or absence. Both scenarios result in poor model performance and variable selection, and hence poor predictive ability [67]. To identify the optimal buffer size, maps were run for all species with buffer sizes of 100 km, 500 km, 1000 km and 1500 km, using 1000 pseudo-absences (see below). These maps were then visually and statistically compared.

The number of pseudo-absences generated in the mapping process can also influence the final mapping product. As a rule of thumb, a proportionally greater number of pseudo-absences to presence data should be used to overcome the possibility that within the randomly selected absences, some may fall on true, yet currently unidentified, presence points [49]. To establish the optimal number of pseudo-absences to use in the mapping, maps for all nine DVS were created and compared using 1:1, 2:1, 5:1 and 10:1 ratios of pseudo-absences to presence data, and additionally using a constant number of 500 and 1000 pseudo-absences for all species. These maps were then visually and statistically compared.

The predictive value of the expert opinion maps were tested using two methods: (i) for each species, 500 pseudo-presences, randomly distributed within the expert opinion species range boundary, were created and run through the BRT mapping alongside 500 pseudo-absences from within the buffer area. Using these pseudo-data, maps were created to identify whether the boundaries given in the expert opinion maps would be recreated by the BRT, such as, for example, the dry area indicated in the north-east of Brazil, identified as too dry for the forest/riverine species *An. darlingi *to survive. (ii) For each species, maps were created using the real occurrence data and, firstly, 500 pseudo-absences randomly selected from the buffer zone and of the same weighting as the true data, and, secondly 500 pseudo-presence points randomly assigned within the expert opinion range but with a weighting half that of the true data plus 500 pseudo-absences created as before. Maps created using both methods were again compared.

All mapping trails were conducted using environmental/climatic variable grids of 5 × 5 km resolution, allowing the production of high quality maps within feasible periods of time.

### Data assembly - bionomics

The need to understand the behaviour and life history characteristics or "bionomics" of the DVS is clear. The impact of interventions, such as ITNs, and control, such as IRS, is closely related to the behavioural characteristics of the local DVS. In addition, such information can help rank the relative importance of a species in an area with regards to transmission and be of major importance to the parameterisation of mathematical models. No recent comprehensive summary of these information exist.

Bionomics information was summarised for each of the nine American DVS (See Additional file 3: Bionomics protocol, for full methodology). A sub-library for each species was created by searching the citations listed within the MAP library by species name. Where a library contained 30 or more citations, it was filtered using the following terms: "behaviour", "behavior", "larva", "biting", "resting" and "habitat", in order to refine the bibliography to include only studies detailing behavioural/bionomic information (Table 2).

**Table 2:** Citation search results for the bionomics survey taken from the MAP database.

Species	Citations (unfiltered)	Citations (filtered)	Citations with data	**References**.
*An. albimanus*	111	68	24	[73,76-91,185,203-209]

*An. Albitarsis*	73	42	15	[74,93,95,96,98-105,140,210,211]

*An. Aquasalis*	30	20	10	[93,100,106-109,111,112,212-214]

*An. Darlingi*	138	78	36	[73-75,90,95,104,113-123,131,141,185,208,210,215-229]

*An. Freeborni*	19	-	3	[127,128,230]

*An. marajoara*	-	-	6	[113,116,117,129,131,132,220]

*An. nuneztovari*	53	28	10	[74,80,95,99,102,116,131,139-141,145]

*An. pseudopunctipennis*	46	32	20	[76,93,109,139,148,149,151-159,208,212,231-234]

*An. quadrimaculatus*	66	36	16	[69,160-171,235-237]

Filter terms were: behaviour, behavior, larva, biting, resting and habitat. *Anopheles albitarsis *and *An. marajoara *libraries were combined prior to data extractions to try and ensure no information was omitted (*An. marajoara *is a sibling of the *An. albitarsis *complex). Articles/data specific to *An. marajoara *were listed in the datasheet separately and those listed under "*An. albitarsis" *are for all other members of the complex, excluding *An. marajoara*

Each of the articles listed in each sub-library was read, and all relevant behavioural information extracted and summarised (Additional file 3: Bionomics protocol). The TAG were consulted and also provided a summary of their knowledge of the bionomics of each DVS which was combined with the relevant literature summary to detail general larval site characteristics (Table 3), known larval habitat types (Tables 4-5), and adult biting and resting behaviour (Table 6).

**Table 3:** Larval site characteristics.

Species	Source	Light intensity		Salinity		Turbidity		Movement		Vegetation	
		
		Helio-philic	Helio-phobic	High (brackish)	Low (fresh)	Clear	Polluted	Still or stagnant	Flowing	**Higher plants, algae *etc***.	No Veg
*An. albimanus*	Summary	3		4	4	1		3	3	12	

*An. albimanus*	TAG	●		○	●	●		●	○	●	

*An. albitarsis*	Summary				1	2	2	1		2	

*An. albitarsis*	TAG	●			●	●	○	●		●	

*An. aquasalis*	Summary	2		3	2	1	1	2	1	3	

*An. aquasalis*	TAG	●		●	●	●		●		●	

*An. darlingi*	Summary	1	6	1	1	2	1	4	4	8	

*An. darlingi*	TAG	○	●		●	●		●	●	●	

*An. freeborni*	Summary										

*An. freeborni*	TAG	●			●	●		●		●	

*An. marajoara*	Summary	2				1	1	1			

*An. marajoara*	TAG	●	○		●	●		●	●	●	

*An. nuneztovari*	Summary	3	3			2	2			1	

*An. nuneztovari*	TAG	●	●		●	●		●	●	●	

*An. pseudopunctipennis*	Summary	2		3	2	2	1	2	1	10	

*An. pseudopunctipennis*	TAG	●		○	●	●	○	●	○	●	

*An. quadrimaculatus*	Summary				2	1	1	2		8	1

*An. quadrimaculatus*	TAG	●			●	●		●		●	

TAG: Rubio-Palis & Manguin (unpub. obs., 2009, 2010), ● = typical, ○ = examples exist. Numbers indicate the number of studies that found larvae under each listed circumstance. *Anopheles albitarsis *refers to the *An. albitarsis *complex, which includes *An. albitarsis, An. albitarsis *sp. B, sp. E and *An. deaneorum. Anopheles marajoara *is listed separately.

**Table 4:** Large larval sites.

Species	Source	Large natural water collections						Large man-made water collections				
		
		Lagoons	Lakes	Marshes	Bogs	Slow flowing rivers	Other	Borrow pits	Rice fields	Fish ponds	Irrigation channels	Other
*An. Albimanus*	Summary	5	1	5	1	1	7		2	1	3	1

*An. Albimanus*	TAG	●	○	●	○			●				

*An. Albitarsis*	Summary	2	1				1		1			

*An. albitarsis*	TAG	●	●	●					●			

*An. aquasalis*	Summary	1		2			4				1	

*An. aquasalis*	TAG	●		●	●				●		●	

*An. darlingi*	Summary	2	2			3	1		1			2

*An. darlingi*	TAG	●			●	●		●			●	○

*An. freeborni*	Summary	1							5			

*An. freeborni*	TAG								●			

*An. marajoara*	Summary						1			1		1

*An. marajoara*	TAG	●	●		●			●	●		●	○

*An. nuneztovari*	Summary	1	1			1				2		1

*An. nuneztovari*	TAG	●	●		●	●		●		●		○

*An. pseudopunctipennis*	Summary	3		1		2	1	1		1	1	1

*An. pseudopunctipennis*	TAG	○				○						

*An. quadrimaculatus*	Summary		4	3			2	1	9			2

*An. quadrimaculatus*	TAG	●	●	●					●		●	

TAG: Rubio-Palis & Manguin (unpub. obs., 2009, 2010), ● = typical, ○ = examples exist. Numbers indicate the number of studies that found larvae under each listed circumstance. *Anopheles albitarsis *refers to the *An. albitarsis *complex, which includes *An. albitarsis, An. albitarsis *sp. B, sp. E and *An. deaneorum. Anopheles marajoara *is listed separately.

**Table 5:** Small larval sites.

Species	Source	Small natural water collections						Small man-made water collections							Artificial sites
		
		Small streams	Seepage springs	Pools	Wells	Dips in the ground	Other	Overflow water	Irrigation ditches	Borrow pits	Wheel ruts	Hoof prints	Puddles near rice fields	Other	**Empty cans, shells *etc***.
*An. albimanus*	Summary	3		1		1	2							4	3

*An. albimanus*	TAG	●		●	●					●	●	●			

*An. albitarsis*	Summary			1										3	1

*An. albitarsis*	TAG	●		●		●				●					

*An. aquasalis*	Summary			2			1							1	1

*An. aquasalis*	TAG	●		●	●				●				●		

*An. darlingi*	Summary	3		5	1		1							1	

*An. darlingi*	TAG	●		●				●		●		●			

*An. freeborni*	Summary						1								

*An. freeborni*	TAG	●		●						●					

*An. marajoara*	Summary	1	1											1	

*An. marajoara*	TAG	●		●		●		●	●	●			●		

*An. nuneztovari*	Summary	2		2		1							3		1

*An. nuneztovari*	TAG	●		●		●				●					

*An. pseudopunctipennis*	Summary	2	2	9		1			1						

*An. pseudopunctipennis*	TAG	●	○	●					○						

*An. quadrimaculatus*	Summary	1		2			2			1				1	2

*An. quadrimaculatus*	TAG	●							●	●					

TAG: Rubio-Palis & Manguin (unpub. obs., 2009, 2010), ● = typical, ○ = examples exist. Numbers indicate the number of studies that found larvae under each listed circumstance. *Anopheles albitarsis *refers to the *An. albitarsis *complex, which includes *An. albitarsis, An. albitarsis *sp. B, sp. E and *An. deaneorum. Anopheles marajoara *is listed separately.

**Table 6:** Adult feeding and resting behaviour.

Species	Source	Feeding habit		Biting habit		Biting time				Pre-feeding resting habit		Post-feeding resting habit	
		
		Anthro-pophilic	Zoo-philic	Exo-phagic	Endo-phagic	Day	Dusk	Night	Dawn	Exo-philic	Endo-philic	Exo-philic	Endo-philic
*An. albimanus*	Summary	2	2	9	2		7	9	0			1	3

*An. albimanus*	TAG	●	●	●	●		●	●		●		●	

*An. albitarsis*	Summary	2	2	4	3		7	3				2	

*An. albitarsis*	TAG	●	●	●	●		●	●		●		●	○

*An. aquasalis*	Summary	1	1	2	2	1	2	1		1		1	

*An. aquasalis*	TAG	●	●	●	●		●	●		●		●	

*An. darlingi*	Summary	12		9	6		15	23	3	1		2	

*An. darlingi*	TAG	●	○	●	●		●	●	●	●		●	

*An. freeborni*	Summary	1	1										

*An. freeborni*	TAG	●	●	●	●		●	●	●	●		●	

*An. marajoara*	Summary	2	2	3			4	1		1		2	

*An. marajoara*	TAG	●	●	●	●		●	●		●		●	

*An. nuneztovari*	Summary	2	4	5	1		3	1		1		2	

*An. nuneztovari*	TAG	●	●	●	●		●	●	●	●		●	

*An. pseudopunctipennis*	Summary	3	2	3				1			1	1	2

*An. pseudopunctipennis*	TAG	●	●	●	●			●		●		●	●

*An. quadrimaculatus*	Summary		3	2			1		1	2		2	

*An. quadrimaculatus*	TAG	●	●	●		○	●	●	●	●		●	

TAG: Rubio-Palis & Manguin (unpub. obs., 2009, 2010), ● = typical, ○ = examples exist. Numbers indicate the number of studies that found adults under each listed circumstance. *Anopheles albitarsis *refers to the *An. albitarsis *complex, which includes *An. albitarsis, An. albitarsis *sp. B, sp. E and *An. deaneorum. Anopheles marajoara *is listed separately.

The bionomics review does not include any detailed information relating to insecticide resistance. This was purposely omitted as this highly dynamic and important aspect of the vectors is being addressed in detail by other groups including the Innovative Vector Control Consortium (IVCC) [68] and groups at the Liverpool School of Tropical Medicine.

## Results

### Data per species

Data were collected in 25 countries in the Americas (Table 7) from which 5408/5942 occurrence points and 2889/3257 sites have been geo-referenced (summary numbers generated in April 2010). "Occurrence" includes all temporal data, where given, and "sites" refers to unique locations. For example, if a study reports the presence of a species every month for a year, this gives 12 incidences of occurrence, but only one geo-referenced site. Data were categorised into the area types that define the size of the area sampled [30]. There were 1509 point locations (≤10 km^2^), 88 wide areas (10-25 km^2^), 42 small polygons (25-100 km^2^) and 369 large polygons (>100 km^2^) (Table 7) in the Americas.

**Table 7:** Number of sites recording species presence per country by area type (points (≤10 km^2^), wide areas (10-25 km^2^), small (25-100 km^2^) and large (>100 km^2^) polygons).

Country	Point		Wide-area	Small polygon	Large polygon
Argentina	19		2	-	-

Belize	228		2	-	13

Bolivia	17		-	1	2

Brazil	304		16	17	136

Colombia	67		4	6	9

Costa Rica	40		-	-	1

Cuba	5		1	-	2

Dominican Republic	5		1	2	-

Ecuador	19		-	-	-

El Salvador	3		-	-	-

French Guiana	7		-	1	-

Grenada	17		1	-	-

Guatemala	51		2	-	-

Guyana	1		-	-	-

Haiti	3		-	-	-

Honduras	1		1	-	-

Mexico	101		11	5	13

Nicaragua	1		1	-	-

Panama	78		-	-	1

Paraguay	2		-	-	-

Peru	49		2	-	13

Suriname	15		6	1	1

Trinidad and Tobago	7		-	-	2

USA	377		34	6	173

Venezuela	92		4	3	3

**Total**	**1509**		**88**	**42**	**369**

Adult collections were reported from 1203 locations, larval collections from 633 and a combination of larval and adult collections from 377 sites. A total of 595 sites reported the presence of more than one DVS, with a maximum of five co-existing species found at San Pedro de Uraba, Colombia. The most popular sampling method of all the reported studies was outdoor landing catches using human bait (MBO). This method was used in 110 studies and at 411 sites. Across the Americas, human landing (indoors and outdoors) sampling techniques were used in 39.3% of studies and at 24.8% of sites compared to the 2.7% of studies and only 1.9% of sites that used animal bait collections. Similar numbers of studies searched for resting mosquitoes inside and outside houses (5.1% resting indoors and 5.2% resting outdoors). However, a greater number of sites were searched for outdoor resting mosquitoes in these studies (16.4% compared to 5.1% resting indoors), although this is a consequence of two large studies that searched for *An. quadrimaculatus *in the USA: Reinert *et al*. [69], 235 sites searched; Seawright *et al*. [70], 148 sites searched. Light trapping or carbon dioxide bait was only used at 4.4% of sites. Morphological identification methods were used at 1102 sites, with PCR techniques applied to identify samples from 178 sites. Longitudinal data were recorded where given, and within these data, 139 studies reported DVS occurrence sampled for over a year, relating to 431 sites. A single sampling period was reported from 85 studies, relating to 1235 point-location sites, whilst 139 studies reported multiple (two or more) sampling periods from 975 point-locations.

*Anopheles darlingi *was reported from the greatest number of geo-positioned point locations (488) and had the greatest number of recorded temporal occurrences (859) (Table 8). In comparison, *An. freeborni *was only reported from 31 site point locations, with only 35 overall occurrence records. The greatest number (377) of point-location sites was recorded from the United States. Guyana had the lowest reported data overall, with only a single point-location reported at the gold-mining town of Mahdia in the Amazon interior, where both *An. aquasalis *and *An. darlingi *were recorded along with a number of other secondary vector species.

**Table 8:** Georeferenced and non-georeferenced occurrence data by species and area type: points (≤10 km^2^), wide areas (10-25 km^2^), small (25-100 km^2^) and large (>100 km^2^) polygons, for the nine American DVS (geographically independent sites (Site) and temporal independent occurrences (Occ)).

	Georeferenced									
	**Point**		**Wide-area**		**Small polygon**		**Large polygon**		**All sites**	

**Species**	**Site**	**Occ**	**Site**	**Occ**	**Site**	**Occ**	**Site**	**Occ**	**Site**	**Occ**

*An. albimanus*	433	822	12	29	7	14	28	38	480	903

*An. albitarsis*	164	482	5	33	13	48	126	131	308	694

*An. aquasalis*	86	162	2	17	10	28	28	28	126	235

*An. darlingi*	488	859	25	67	15	106	124	153	652	1185

*An. freeborni*	31	35	7	28	1	1	5	6	44	70

*An. marajoara*	55	91	4	4	3	4	7	22	69	121

*An. nuneztovari*	199	433	11	34	14	14	116	121	340	602

*An. pseudopunctipennis*	285	454	7	19	6	6	15	16	313	495

*An. quadrimaculatus*	356	542	27	30	6	22	168	509	557	1103

**Total**	**2097**	**3880**	**100**	**261**	**75**	**243**	**617**	**1024**	**2889**	**5408**

	**Non-georeferenced**									

	**Point**		**Wide-area**		**Small polygon**		**Large polygon**		**All sites**	

**Species**	**Site**	**Occ**	**Site**	**Occ**	**Site**	**Occ**	**Site**	**Occ**	**site**	**Occ**

*An. albimanus*	44	109	5	7	2	2	2	2	55	125

*An. albitarsis*	24	28	0	0	0	0	1	1	25	29

*An. aquasalis*	0	0	0	0	0	0	1	1	1	1

*An. darlingi*	117	120	0	0	1	1	1	1	122	132

*An. freeborni*	0	0	0	0	0	0	0	0	0	0

*An. marajoara*	7	7	0	0	0	0	0	0	7	7

*An. nuneztovari*	93	93	0	0	0	0	1	1	96	97

*An. pseudopunctipennis*	29	32	6	42	1	1	2	2	38	77

*An. quadrimaculatus*	21	63	2	2	0	0	0	0	24	66

**Total**	**335**	**452**	**13**	**51**	**4**	**4**	**8**	**8**	**368**	**534**

### Mapping trials

All results for each mapping trial are given in Additional file 4: Summary tables showing evaluation statistics for all mapping trials and final BRT environmental and climatic variable selections for the final, optimal predictive maps. Optimal mapping categories were evaluated visually and using the deviance and AUC statistics, with the caveat that these could only be used as a guide rather than a true indication of predictive performance, as different data sets were used for each map produced.

The optimal buffer size within which pseudo-absences were randomly selected was determined to be 1000 km, with five out of nine species maps judged to perform better at this buffer size than those produced using the alternative sizes. A ratio of 5:1 pseudo-absences to presence data was applied, taking into account 250 (500 half weighted) pseudo-presences. This level of pseudo-absence input provided enough data to counteract any potential interference that may have been caused by the random inclusion of unidentified presences from within the absence data.

The EO mapping test indicated that where random pseudo-presences are created within the EO range, and no real occurrence data were included, the model would predict a high probability of presence within the whole expert opinion range and calculate a high deviance value for all species, indicating overall, a poor predictive performance. Where the hybrid method was used that incorporated both real occurrence data plus 500 half-weighted pseudo-presence points randomly assigned within the EO range, the mapping performance was greatly improved. Maps created using only the real presence data, produced a low deviance value, but visually, predictive performance was judged to be poor, possibly due to a paucity of data for some species. As with all species mapping, occurrence data will be biased to areas that are accessible for sampling, or as succinctly stated by Coetzee [71], reflect the distribution of entomologists rather than mosquito species. As such, all occurrence-based mapping and mapping evaluations must be treated with some level of caution, especially where the data is sparse. It was thus considered that the hybrid maps performed better overall and are presented here.

### Predictive maps

The BRT species maps for all nine American species are given in Additional file 2: Predictive species distribution maps for the nine DVS of the Americas, and are summarised in Table 9. Spatial constraints prevent all the species being discussed in full here, however *Anopheles darlingi*, considered one of the most important malaria vectors in the Neotropical Region [23], is reviewed below.

**Table 9:** Evaluation statistics and the top five environmental/climatic variables selected by the BRT for the nine DVS in the Americas using presence data plus 500 pseudo-presences generated from within the EO boundary (weight = 0.5) and a 1000 km buffer area for generation of 5:1 pseudo-absence:presence at a 5 km resolution.

Species	Evaluation		Environmental variables	
*An. albimanus*	Deviance:	0.2739	1	LST (P1)
	Correlation:	0.8278	2	DEM
	Discrimination (AUC):	0.9683	3	Prec (P1)
	Kappa:	0.7672	4	LST (mean)
			5	LST (max)

*An. albitarsis*	Deviance:	0.2919	1	Prec (A1)
	Correlation:	0.8207	2	Prec (mean)
	Discrimination (AUC):	0.9613	3	DEM
	Kappa:	0.7643	4	LST (A1)
			5	NDVI (P1)

*An. aquasalis*	Deviance:	0.3944	1	DEM
	Correlation:	0.7458	2	Prec (P1)
	Discrimination (AUC):	0.9443	3	Prec (A2)
	Kappa:	0.6386	4	LST (P1)
			5	LST (max)

*An. darlingi*	Deviance:	0.2763	1	Prec (max)
	Correlation:	0.8351	2	LST (max)
	Discrimination (AUC):	0.9684	3	Prec (mean)
	Kappa:	0.7902	4	LST (P2)
			5	Prec (P2)

*An. freeborni*	Deviance:	0.2932	1	Prec (P1)
	Correlation:	0.8191	2	Prec (A1)
	Discrimination (AUC):	0.9666	3	Prec (max)
	Kappa:	0.7573	4	DEM
			5	LST (A1)

*An. marajoara*	Deviance:	0.3354	1	Prec (P2)
	Correlation:	0.7962	2	Prec (A1)
	Discrimination (AUC):	0.9482	3	LST (P2)
	Kappa:	0.7219	4	NDVI (A1)
			5	Prec (min)

	Deviance:	0.3094	1	Prec (max)
*An. nuneztovari*	Correlation:	0.8161	2	Prec (mean)
	Discrimination (AUC):	0.9555	3	LST (mean)
	Kappa:	0.7633	4	Prec (A1)
			5	LST (min)

*An. pseudopunctipennis*	Deviance:	0.3567	1	LST (P1)
	Correlation:	0.7682	2	DEM
	Discrimination (AUC):	0.9432	3	Prec (P1)
	Kappa:	0.6768	4	MIR (mean)
			5	MIR (P1)

*An. quadrimaculatus*	Deviance:	0.1237	1	Prec (min)
	Correlation:	0.9271	2	Prec (mean)
	Discrimination (AUC):	0.9904	3	Prec (P1)
	Kappa:	0.9080	4	LST (A1)
			5	DEM

**Figure 1:**
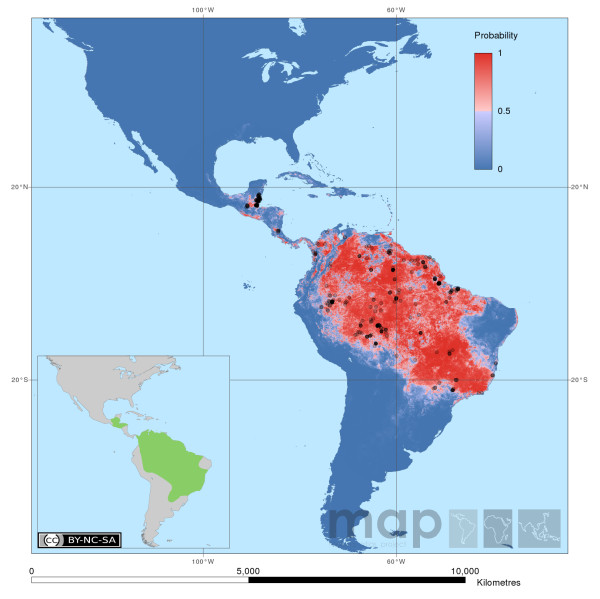
**Map details: The predicted distribution of *An. darlingi *mapped using hybrid data (318 occurrence data plus 500 pseudo-presences weighted at half that of the occurrence data and randomly selected from within the Expert Opinion (EO) range)**. Pseudo-absences (2840) were generated at a ratio of 5:1 absence to presence points, taking into account 250 pseudo-presence points (500 at half weight), and were randomly selected from within the 1000 km buffer surrounding the EO (EO shown in the inset map). Predictions are not shown beyond the buffer boundary. The black dots show the 318 occurrence records for *An. darlingi*. **Map statistics**: Deviance = 0.2763, Correlation = 0.8351, Discrimination (AUC) = 0.9684, Kappa = 0.7902. **Environmental variables**: 1. Prec (max), 2. LST (max), 3. Prec (mean), 4. LST (P2), 5. Prec (P2) (Please see Additional file 4 for abbreviations and definitions). **Copyright**: Licensed to the Malaria Atlas Project [197] under a Creative Commons Attribution 3.0 License. **Citation**: Sinka *et al*. (2010) The dominant *Anopheles *vectors of human malaria in the Americas: occurrence data, distribution maps and bionomic précis, *Parasites and Vectors *2010, **3**:72.

*Anopheles darlingi *is a lowland, riverine, forest dwelling species (see below and Table 4), unable to survive in dry climates, including for example, north-eastern Brazil (Rio Grande de Norte, Paraiba *etc*.) as is approximated in the EO map (Figure 1, inset). Figure 1 shows the predicted species distribution using hybrid data (318 occurrence data plus 500 pseudo-presences weighted at half that of the occurrence data and randomly selected from within the EO range) and a 5:1 ratio of pseudo-absence to presence data, taking into account the pseudo-presence points. Also indicated are the environmental variables identified by the BRT as being the most influential amongst those applied to the model in describing the species distribution.

Maximum precipitation is the variable identified as having the highest relative influence (36.31%) on the presence of *An. darlingi *compared to 9.04% for the second-rated variable (maximum LST) (Figure 1; Table 9). Precipitation is selected twice more within the top five (third: mean; and fifth: phase of the bi-annual cycle) and LST was selected once more (fourth: phase of the bi-annual cycle). Aside from precipitation and LST, the only other two variables selected within the top ten list were elevation (DEM) at seventh place with a relative influence of only 3.61% and MIR (mean), listed tenth and with a relative influence of 3.04%. Land use appears to have little effect, with Globcover channel 160 (closed to open (>15%) broadleaved forest regularly flooded (semi-permanently or temporarily), fresh or brackish water) only listed as the thirteenth variable with a relative influence of 1.87%.

The variables selected by the BRT correspond well to the bionomics of *Anopheles darlingi*. Larvae of this species are most often found in slow flowing rivers, associated with floating debris [72-74]. Rain will impact on river current and height, potentially flushing away the larvae and the river debris they inhabit or causing them to become stranded once the rain lessens or stops and the river recedes [73,75]. Land Surface Temperature does not have such a clear cut impact on *An. darlingi *and could be influencing its distribution in a number of ways. For example, it may be indicating the presence of hotter, dryer environments away from the forests, such as those that limit the distribution of *An. darlingi *in the north east of its range. Alternatively, LST may be identifying the higher temperatures associated with its lowland range, although there is recent evidence that this species is able to survive at higher altitudes than previously suspected (see below).

### Bionomics

#### *Anopheles albimanus*

The larval sites used by *An. albimanus *are characterised across its range as open, sunlit and containing clear water [76] (Table 3). The species can be found in natural and man-made habitats where these characteristics exist. For example, it occurs in recently planted rice fields, or in older fields with sunlit areas in between the rice plants [77] (Table 4). *Anopheles albimanus *has been associated with floating mats of blue-green algae [76,78,79], which are often found in sunlit waters. The larvae of this ubiquitous species tolerate a wide variation in water chemistry and are able to exploit diverse food sources [78], enabling them to survive in both fresh water (*e.g*. irrigation channels, small ponds, marshes, slow flowing streams and river margins [73,80-83]) and brackish water (*e.g*. mangrove swamps [78,83,84]) (Tables 3-5).

*Anopheles albimanus *is predominantly exophagic with exophilic resting behaviour [77,80,85,86] (Table 6), however there is some indication that in the northern reaches of its distribution (Mexico, Central America), this species exhibits a preference for resting indoors after feeding [87,88]. In a mark-recapture study that ultimately influenced changes in the vector control regime in southern Mexico [87], Bown *et al*. [89] examined *An. albimanus *resting behaviour and found that 80% landed indoors after feeding. *Anopheles albimanus *bites in the evening and during the night [77,80,85,86,90,91]. It appears to show a tendency for zoophily, but this is dependent upon location [86,89,91,92]. In Colombia, Solarte *et al*. [86] described *An. albimanus *as exhibiting "a high degree of anthropophilic activity". In contrast, Loyola *et al*. [92] described this species as highly zoophilic in Mexico, however they also pointed out that host availability and other ecological conditions influence the host choice of this species.

#### *Anopheles albitarsis *complex

The *An. albitarsis *species complex includes *An. albitarsis *(formerly Sp. A), *An. albitarsis *Sp. B and Sp. E, *An. marajoara *(formerly Sp. C) and *An. deaneorum *(formerly Sp. D). *Anopheles marajoara *is discussed separately below. Overall, members of this complex exhibit larval habitat preferences similar to *An. albimanus*, occupying sunlit, clear, fresh water [93,94] (Rubio-Palis, unpub. obs.) (Table 3), although there are examples of it being found in atypical conditions, Da Silva-Vasconcelos *et al*. [95] found relatively high numbers, compared to other species, in brick pits containing turbid water mixed with clay particles [95]. Typical habitats are fresh, still water bodies such as lagoons, lakes or rice fields [74,93,96] (Table 4). Indeed, members of the *An. albitarsis *complex are closely associated with human rice cultivation [97,98], although, like *An. albimanus*, immature stages tend to be found only in fields with early stages of rice growth [98]. Adults are generally exophilic in their resting behaviour [99,100], but will bite both indoors and outdoors and appear to show little host preference, biting humans and animals indiscriminately (dependent on location), during the evening and at night [95,99-105] (Table 6).

#### *Anopheles aquasalis*

Grillet [106] described *An. aquasalis *as an opportunistic species whose individuals "may be poor competitors or may develop few anti-predator defences". In areas where *An. aquasalis *and *An. albimanus *co-exist, *An. albimanus *will dominate (Rubio-Palis, unpub. obs.) suggesting that the inability of *An. aquasalis *to out-compete other dominant *Anopheles *species may be highly influential in defining its ecological requirements (as its name implies, it is generally only found in coastal areas).

*Anopheles aquasalis *is found in sunlit habitats containing emergent vegetation, in both brackish and fresh water [93,106,107] (Table 3). It is considered to "prefer" clear, still, non-polluted water such as stream pools, mangrove swamps, grass swamps, lagoons and ditches [108,109], although there are examples of it being found in turbid, slow flowing water bodies, in relatively high numbers (*e.g*. [93]) (Tables 4 &5).

Adults, once again, are opportunistic, feeding indoors or outdoors on animal or human hosts, but generally resting outdoors before and after feeding [100,110-112]. Biting tends to begin at dusk, peaking early in the night, and tailing off as the night progresses [111,112] (Table 6). The time of the biting peak and bias towards endo-or exophagy depends on the location, for example in Maranhão, Brazil, Xavier & Rebelo [111] observed a tendency to bite at dusk, indoors. Berti *et al*. [112] measured biting behaviour in two villages in Sucre State, Venezuela, where *An. aquasalis *predominantly bit outdoors, and found that biting peaked earlier (7 pm) in the village of Guayana, where greater numbers of *An. aquasalis *were found, than in Santa Fe, where the peak occurred between 8 and 9 pm. During the study, two females were collected biting during the day.

#### *Anopheles darlingi*

*Anopheles darlingi *is considered to be one of the most efficient malaria vectors in the Neotropical region [23]. It is mainly a riverine mosquito, generally confined to rural, lowland forested locations [113] (Rubio-Palis & Manguin, unpub. obs.). Conversely, however, Vittor *et al*. [114] suggest that deforestation and human environmental alteration can create habitats which are favourable to *An. darlingi *as they found higher densities in areas with limited forest cover than in areas predominated by forest. The larval habitats of *An. darlingi *can be characterized as: natural water bodies such as lagoons, lakes and particularly slow flowing streams or rivers with shaded, clear water, and associated submersed vegetation such as bamboo roots from overhanging spiny bamboo [72-74,113] (Tables 3-5). Larvae are encountered most frequently in patches of floating debris along river margins [73]. There are examples of larvae being found in uncharacteristic locations, however, such as in slightly brackish water (Belize, [73]), in low numbers in turbid, polluted water (brick pits, [95]) and in abandoned gold mine dugouts in southern Venezuela [115], further suggesting a level of adaptation to areas altered by humans [113].

*Anopheles darlingi *tends to rest outdoors regardless of where it has taken its blood meal [116,117] (Table 6). Adults will bite throughout the night [95,117-120] and the degree of endo- and exophagy of this species varies from one place to another as does its host preference [121] (Table 6). It has been suggested that the biting pattern of *An. darlingi *may represent an adaptation to human behaviour [117,121]. Moreno *et al*. [117], for example, contend that the all night activity of *An. darlingi *in the gold mining areas of southern Venezuela is a response to the all night activity of the miners. Furthermore, a number of studies that report exophagy in this species (Table 6) refer to peri-domestic sampling [113,116,122] at sites where indoor insecticide spraying is or had recently been used for vector control [113,117,122,123]. Other indications of the adaptability of *An. darlingi *are emerging, with an ongoing study in Venezuela, close to the Brazilian border (Roraima), recently reporting that specimens have been collected at altitudes above 800 m (Rubio-Palis, unpub. obs: re. Berti *et al*. at the Instituto de Altos Estudios "Dr. Arnoldo Gabaldon", Ministry of Health).

#### *Anopheles freeborni*

Studies examining the bionomics of *An. freeborni *tend to have been performed prior to 1985 limiting the amount of useful data found within the MAP library. Therefore additional searches of older references were needed to develop a more complete picture of the bionomics of this species.

McHugh [124] suggests that *An. freeborni *is an opportunistic feeder. In a study where its blood meals were identified, host availability was key in determining blood source. For example, the *An. freeborni *females collected in cattle areas contained the highest proportion of bovine blood, whereas 35% had fed on dogs at sites of human habitation near a rice production area, and no bovine blood was found. This suggests that the species is zoophilic, although a preference is not indicated in this study. Washino & Tempelis [125], in a similar study, reported very low levels of human blood in *An. freeborni *(<1% of mosquitoes tested) even though sampling was conducted in areas of human habitation. They also indicated the opportunistic nature of this species, given the high level of rabbit blood meals found. However, once again, the proportion was highly dependent on rabbit availability. The resting mosquitoes were collected from farmyard sheds, abandoned domestic animal shelters, house porches, artificial shelters, bridges and culverts, indicating an outdoor resting preference.

Orr & Resh [126] demonstrated a positive association between *An. freeborni *larval densities and plant cover, indicating that, as with all the other DVS in the Americas, vegetation is a key characteristic of their larval habitats. This is reflected by the species' ready utilization of rice fields, although significantly higher numbers of adult *An. freeborni *are found in riparian and mixed habitats than in rice and pasture habitats [127,128] (Table 4).

#### *Anopheles marajoara*

*Anopheles marajoara *is a member of the *An. albitarsis *complex. Previously believed to be a secondary, local vector of minor importance, it was identified as a DVS in the 1990s in a study conducted in Amapa, Brazil [129], where it was found in high densities and with high levels of *Plasmodium *infection when compared to *An. darlingi*. Here, therefore, this species is considered separately from other members of the *An. albitarsis *complex.

*Anopheles marajoara *is a lowland species, associated with wetlands, secondary forests and human intervention [115,129] (Rubio-Palis, unpub. obs.). Moreno *et al*. [117] suggest that the recent studies in Brazil identifying *An. marajoara *as dominant over *An. darlingi *may be a result of human interventions which favour species that oviposit in open lagoons with abundant macrophytes. Conn *et al*. [129] described forest clearance and pollution as reducing the availability of larval sites for *An. darlingi *whilst increasing the availability of sites such as agricultural ponds and sunlit marshy areas, which are the preferred habitats of *An. marajoara*. Overall, *An. marajoara *larval sites are generally sunlit with clear, still water, although there are examples of this species being found in both clear and muddy waters, such as fish ponds [113] and gold mine dugouts [115] (Tables 3-5).

In Amapa, *An. marajoara *is described as exclusively exophilic [129], which appears to be the case across its range [99,116,117] (Rubio-Palis, unpub. obs.), however a study in Colombia examining the indoor resting behaviour of anophelines, reported *An. marajoara *resting indoors, close to the ground [130], suggesting some endophilic behaviour in limited areas (Table 6).

*Anopheles marajoara *bites both humans and animals [102,116,131], both indoors and outdoors throughout the night, with biting tending to peak in the evening [99,113,117,129] (Table 6), though again these characteristics can vary according to location. For example, a study in southern Venezuela reported marked exophagic behaviour, with 74.2% of *An. marajoara *captured biting outdoors [117]. This contrasts with a study carried out in western Venezuela [99] that reported indiscriminate indoor and outdoor biting. There is currently some debate, however, about the identity of members of the *An. albitarsis *complex in Venezuela. Past investigations have only identified the presence of *An. marajoara *[117,132], but a recent study suggests the possibility of misidentification and the additional presence of *An. janconnae *[133]. A consensus for this "discovery" has yet to be reached.

#### *Anopheles nuneztovari*

The *An. nuneztovari *species complex is yet to be fully resolved and there is a need for some clarification of sibling identity. The complex contains either two or three cytological species (A and B/C), with B and C possibly two forms of a single species [134-137] (Rubio-Palis & Manguin, unpub. obs.). Furthermore, Calado *et al*. [137] have recently formally resurrected *An. goeldii *from synonymy with *An. nuneztovari *A, making the task of truly categorising members of this complex all the more challenging.

*Anopheles nuneztovari *larvae are found in both sunlit and shaded habitats [74,138] (Rubio-Palis, unpub. obs.). Sites usually contain fresh, clear, still or flowing water with floating or emergent vegetation [74,138] (Table 3). Despite this, Nagm *et al*. [74] report finding this species in several turbid water bodies, and da Silva-Vasconcelos *et al*. [95] found small numbers in brick pits containing very turbid water polluted with brick dust. Indeed, Service [28] described *An. nuneztovari *larval habitats as "muddy waters of pools, vehicle tracks, hoof prints, small ponds, especially in and around towns".

Habitats are found in small or large, natural or constructed bodies of water, including lagoons, lakes, slow flowing rivers, fish ponds, gold mine dugouts, rain puddles and temporary or permanent pools [80,138-140] (Table 4 &5). Tadei & Thatcher [141] described *An. nuneztovari *as a species readily able to colonise and even dominate in altered environments and yet, despite this characteristic, it is not known to breed in rice fields.

Adult behaviour differs depending on the sibling species, most specifically in terms of their biting times, with *An. nuneztovari *A (Brazil) biting earlier, peaking between 6 and 8 pm [95,141,142], and *An. nuneztovari *B/C (Venezuela and Colombia) biting later and throughout the night, peaking between 10 pm and 2 am [142]. Olano *et al*. [80] reported peri-domiciliary biting of *An. nuneztovari *early in the evening in Buenaventura, Colombia, however this may be the result of low biting densities rather than a true indication of preference. The Brazilian sibling is considered to be a non-vector, possibly due to its behaviour rather than of an inability to transmit malaria [142,143], given that there is evidence of *Plasmodium *infection in *An. nuneztovari *in Amapa, Brazil [144,145].

Service [28] suggested that *An. nuneztovari *mainly feeds on animals, but will bite humans outdoors. Studies in Amapa, Brazil and in Venezuela, which analysed blood meals of *An. nuneztovari*, suggest zoophilic behaviour, but an accompanying human landing catch in the same area found over 120 mosquitoes/person/night [99,102,116]. Exo- and endophagy of members of this species complex vary with location but Tadei & Thatcher [141] suggested that human behaviour, such as a propensity to stay outdoors late into the evening, or the application of insecticides, may influence biting location. The majority of studies summarised here report exophagic behaviour [80,99,116,139,141] (Table 6), with only Rubio-Palis & Curtis [99] reporting both endo- and exophagic behaviour in western Venezuela. Rubio-Palis & Curtis [99] mentioned the contrasting observations of exo- and endophagy reported for *An. nuneztovari *and suggested this may be due to collection bias (collector, location or short series of observations) or a result of different behavioural patterns between sympatric siblings, which again highlights a need for further clarification of species identity. All sibling species within the *An. nuneztovari *complex are highly exophilic, resting outdoors both before and after feeding [99,116] (Table 6).

#### *Anopheles pseudopunctipennis*

*Anopheles pseudopunctipennis *is a complex of at least two species [146,147] and two forms [148] (Rubio-Palis & Manguin, unpub. obs.). It can survive and transmit malaria at altitudes higher than many other DVS, with its range extending up to approximately 3000 m [22,149] (Rubio-Palis & Manguin, unpub. obs.). This species is most frequently found in sun-exposed, shallow, clear and freshwater streams or river pools with abundant filamentous algae [149], although there are a number of reports of larvae found in turbid, cloudy water [93,109,149], including at one site polluted with cow faeces [149]. The majority of larval habitats have fresh water, but about 10% contain brackish or sea water [93,149] (Table 3). Past studies conducted in Grenada suggested that *An. pseudopunctipennis *was restricted to still or stagnant water [150] but more recent investigations on this island indicate that it can survive in slow flowing water bodies, possibly protected against the current by mats of *Spirogyra*-type green filamentous algae [109,149]. The presence of such filamentous algae is a key characteristic associated with larval habitats of this species [93,109,149,151-154] (Table 3). Indeed, a study that examined the potential impact of *An. pseudopunctipennis *control *via *environmental manipulation demonstrated significant reductions in densities after the removal of filamentous algae from larval sites [151].

Service [28] stated that adult *An. pseudopunctipennis *"...feed almost indiscriminately on humans and domestic animals, indoors or outdoors...". Studies conducted in southern Mexico corroborate his statement, for example Fernandez-Salas *et al*. [155] found that a greater proportion of *An. pseudopunctipennis *were attracted to horse-baited traps than to humans (although significant numbers were captured on humans), however in a previous study in the same four villages [156], they found that a high proportion of the *An. pseudopunctipennis *females resting indoors contained human blood. They suggested that host availability was responsible for host selection, rather than this being due to any preference exhibited by the mosquitoes. Lardeux *et al*. [157], in a study specifically designed to examine host preference, found some level of choice exhibited by females. They suggested that this species is not highly anthropophilic, but reiterated its opportunistic nature, stating that *An. pseudopunctipennis *females will bite the first "preferred" host they encounter (in their experiment the feeding preference was ranked as: sheep, goats and donkeys, followed by humans and cows).

Service [28] also stated that "[*An. pseudopunctipennis*] rest outdoors after feeding", however the studies above [155,156], and those of Casas *et al*. [158], who used the mark-recapture method to identify resting behaviour of fed and unfed mosquitoes, indicated that a proportion of *An. pseudopunctipennis *will rest indoors both before and after feeding. However, Loyola *et al*. [159] suggested that IRS with DDT has not only increased insecticide resistance in some areas, but also promoted more exophilic behaviour.

*Anopheles pseudopunctipennis *bites during the night, with small variations in peak activity depending on location and host, for example Fernandez-Salas *et al*. [155] demonstrated a uni-modal peak, with indoor biting peaking at 1 am and outdoor biting peaking slightly earlier, at midnight. Interestingly, they also reported a bimodal biting pattern on horse bait, where biting peaked at 7 pm, with a second, smaller peak occurring between midnight and 1 am (Table 6).

#### *Anopheles quadrimaculatus *subgroup

The Quadrimaculatus Subgroup of the Maculipennis Group is often mis-reported as a complex (*e.g*. [19,69,70]). Within this subgroup, *An. quadrimaculatus *(formerly Sp. A) is the most wide-spread species [19,70], considered the most "important" [70], and it is the only species within the subgroup identified as a DVS [25-29]. Therefore only this species is given further consideration here, with the caveat that some studies included in the bionomics review do not distinguish species and report *An. quadrimaculatus s.l*. However, as suggested by Seawright *et al*. [70], owing to its abundance, seconded only by *An. smaragdinus *(formerly *An. quadrimaculatus *Sp. B), *An. quadrimaculatus *(and *An. smaragdinus*) "are probably the species that most researchers have studied in the past".

*Anopheles quadrimaculatus *is highly associated with rice cultivation [69,160-166] (Table 4), showing a preference for the oligotrophic conditions found when the rice fields are first flooded [162]. Such conditions reflect those found in the natural larval habitat of this species: generally fresh, still water in relatively large sites such as lakes and marshes with emergent vegetation [69,167-170] (Tables 3 &4). Unusual larval sites have been reported including a sewerage retention pond containing highly polluted effluent draining from a pig farm, a small plastic bucket containing rainwater, submerged leaf litter and floating pine needles [171], and wastewater evaporation-percolation ponds in Florida [172] (Table 5).

Adults appear to be generally zoophilic, biting and resting outdoors [69,168,170] (Table 6), however, amongst all studies summarised, this may be an artefact of the areas sampled and the lack of human hosts rather than an indication of preference [168,170]. Of the species in this subgroup, however, *An. quadrimaculatus *appears to show the highest level of anthropophily. Jensen *et al*. [168] examined blood meals in *An. quadrimaculatus *(Sp. A), Sp. B (*An. smaragdinus*) and Sp. C_1 _(*An. diluvialis*) at a campsite where human hosts were available, and in a woodland approximately a mile away, where there was a lower chance of human contact, and found that 10.7% of *An. quadrimaculatus *sampled at the campsite had fed on humans compared to none in the wooded area. The other two species demonstrated very low or no human biting at both sites. Reinert *et al*. [69], in a comprehensive description of the taxonomy and bionomics of the Quadrimaculatus Subgroup, also reported such exophagic and zoophilic behaviour, describing frequent collections of engorged females from horse stables and cattle barns and a number of observations of feeding on large domestic animals.

*Anopheles quadrimaculatus *bites throughout the night, showing higher activity at dusk and dawn [69]. Resting behaviour, as with all other DVS described here, is exophilic, and includes sites in holes and rot cavities in trees, livestock barns, outdoor latrines, under bridges and under the eaves of buildings [69,170,173,174] (Table 6).

### Secondary vectors

This study has focused on describing the distribution and bionomics of the main malaria vectors in the Americas. However, there are numerous secondary or local vectors that may also play an important yet often forgotten role in malaria transmission. In the Americas, secondary vectors include the various species of the subgenus *Kerteszia *(*e.g*. *An. cruzii, An. bellator *and *An. neivai*), whose larvae characteristically inhabit water contained in bromeliads, as well as *An*. (*Anopheles*) *vestitipennis, An*. (*Ano*.) *neomaculipalpus, An*. (*Nyssorhynchus*) *braziliensis, An*. (*Nys*.) *triannulatus, An*. (*Nys*.) *strodei, An*. (*Ano*.) *intermedius *and members of the *An*. (*Nys*.) *oswaldoi *complex. Their "secondary" vector status is a factor of location, distribution, vectorial capacity and, occasionally, history (*i.e*. a species once considered primary, but now relegated to a secondary role through changes in the local environment). Circumstantial evidence, or the identification of *Plasmodium *circumsporozoite proteins in females, can lead to a species being incriminated as a vector, where in reality it may have little to no impact [23]. However, some "secondary" species are proven and potent vectors within their local range.

Both *An. cruzii *and *An. bellator *are identified by White [29] as main malaria vectors, yet their larval habitats (bromeliads) restrict their role in malaria transmission to areas where such plants are abundant, for example in the rainforests of Brazil where these two species are considered to be primary local vectors. Deforestation may have reduced the availability of habitats for these species but they ought not to be overlooked as their range extends along the eastern coast of South America, from Guyana to the southernmost tip of Brazil. Moreover, there are reports of these species being found in bromeliads and artificial containers in urban and peri-urban sites [175,176] with *An. cruzii *specifically described as an aggressive biter throughout the day and night [177]. *Anopheles neivai *also makes use of bromeliads during its larval stage and is an important vector of human malaria in the Pacific coastal areas of Colombia [178,179]. It has been responsible for outbreaks of malaria in the Venezuelan Andes at altitudes above 1000 m [180,181].

*Anopheles vestitipennis *is considered to be a secondary vector species of major importance within its range. In Belize, it has been described as a primary vector, ousting *An. albimanus *which plays a more secondary role there [182-185]. Achee *et al*. [183] found it to be positive for both *P. falciparum *and *P. vivax *and Loyola *et al*. [186] found it to be the most abundant species (>80% of those collected on human bait) in their study area within the Lacandon rainforest of Chiapas, Mexico, and the only species in the area to be positive for the *P. vivax *antigen. They also demonstrated that it readily bites humans both indoors and outdoors. *Anopheles vestitipennis *is also found in the Caribbean islands where it may have been involved in malaria outbreaks in Cuba [187] and Haiti (Rubio-Palis, unpub. obs.).

Malaria parasites have also been detected in several other species. De Oliveira Ferreira *et al*. [188] found natural infections of *Plasmodium *in *An. triannulatus, An. braziliensis, An. strodei *and *An. oswaldoi *in Rondonia, Brazil. *Anopheles triannulatus *and *An. strodei *were only infected with *P. vivax*, however the very small sample sizes (the largest being five specimens of *An. triannulatus*), prevents the drawing of any conclusions regarding any refractory characteristics.

*Anopheles oswaldoi *has been confirmed as a malaria vector in Brazil [119,188-190], and it is the principal vector in the State of Acre in the Brazilian Amazon where it has been found in large densities and with relatively high sporozoite rates for *P. falciparum *(3.41%), *P. vivax*-210 (2.26%), *P. vivax*-247 (1.22%) and *P. malariae *(0.42%) [189]. *Anopheles oswaldoi *has also been incriminated as a vector of *P. vivax *in Colombia [191], Peru [192] and Venezuela [193].

The role of *An. neomaculipalpus *in malaria transmission is not clear but it has recently been found to be positive for *P. vivax *in Venezuela [194,195] and positive for *P. falciparum *in Colombia [195,196] and it is considered to be a highly anthropophilic species ([195]). Moreno *et al*.[195] suggested that this species may play a role as a secondary vector of "frontier malaria" in areas of forest subject to recent human activity that can increase vector diversity with the creation of new larval sites. De Oliviera Ferreira *et al*. [188] reiterated this hypothesis and suggested that the presence of infection amongst these secondary vectors may be linked to the extensive environmental changes that may reduce the populations of sylvatic animals in an area whilst simultaneously increasing the number of humans, which will inevitably cause a higher level of vector-human contact.

## Discussion

### Reliability of the predictive maps

The predictive maps presented here are unlikely to be perfect representations of the full distributions of the DVS. The model has been applied to species presence data from one of the largest, most comprehensive, contemporary databases of DVS occurrence available, yet the limitations of these opportunistic data for species mapping are evident. Biases in collection location, limited data for some species and a lack of consistency in sampling methodology all contribute to modelling and output uncertainty. Despite these caveats, the maps represent the first attempt to model DVS distributions in the Americas using extensive occurrence data, contemporary EO range maps and a consistent methodology across all species. Previous maps have either focussed on a single species or subgroup [19], single countries [20,21], been based solely on EO [22,23], or, specimens collected over a large period of time [18]. The maps are best considered a starting point in a continuing process of describing and understanding DVS distributions, and not as an end product. Therefore, and in accordance with the open access principles of the MAP, all the data compiled here will be made freely available on the MAP website [197] and efforts made to improve the maps as more data become available.

### Bionomics

The bionomics review highlights DVS behaviour and life-history characteristics that are relevant for mosquito control, but also clearly indicates the marked behavioural plasticity of each species. The influence of human behaviour such as insecticide use, environmental disturbance to a greater or lesser extent, or host activities in the evening and night also drive local variation in species bionomics. Moreover, concerns regarding species identity also add to the uncertainty in categorising species behaviour and thus local, expert knowledge must be consulted when interpreting or acting on the data summarised here.

### Future work

This is the first in a series of three publications describing the distribution and relevant bionomics of the global DVS of *P. falciparum*. The remaining two publications will detail the DVS of Africa, Europe and the Middle-East (Sinka *et al*: The dominant *Anopheles *vectors of human malaria in Africa, Europe and the Middle East: occurrence data, distribution maps and bionomic précis, unpublished), and the DVS of the Asian Pacific region (Sinka *et al*: The dominant *Anopheles *vectors of human malaria in the Asia Pacific region: occurrence data, distribution maps and bionomic précis, unpublished). Together, these three publications are intended to provide a baseline set of data and maps and summarise the current knowledge of the bionomics of the 41 species (DVS) identified as the primary vectors of *P. falciparum *and *P. vivax *malaria.

## Conclusions

Species distribution mapping is a dynamic process. The advent of new modelling techniques [47], high resolution climatic and environmental spatial data at local and global scales [52-55,57,58,198], increasing computer processing capacity and a greater on-the-ground knowledge of what drives and limits the range of a species provide the tools for increasingly accurate map production. These maps are often limited by the data available to the model rather than the modelling process itself. Increasing openness and a willingness to share data across disciplines and groups will prevent duplication of effort in data-rich areas, and encourage the development of systematic vector sampling procedures in areas where the information is relatively poor.

## List of abbreviations

DVS: Dominant Vector Species; BRT: Boosted Regression Trees; IVM: Integrated Vector Management; MIMP: Mosquito Information Management Project; TAG: Technical Advisory Group; MAP: Malaria Atlas Project; WRBU: Walter Reed Biosystematics Unit; EO: Expert Opinion; GIS: Geographic Information System; PCR: Polymerase Chain Reaction; ITNs: Insecticide Treated Bednets; IRS: Insecticide Residual Spraying; MES: Marianne E; Sinka AUC: Area Under the operating characteristic Curve; TFA: Temporal Fourier Analysis; SRTM: Shuttle Radar Topography Mission; DEM: Digital Elevation Model; MODIS: MODerate Resolution Imaging Spectroradiometer; NASA: National Aeronautics and Space Administration; AVHRR: Advanced Very High Resolution Radiometer; NDVI: Normalized Difference Vegetation Index; LST: Land Surface Temperature; MIR: Middle Infrared Radiation; MERIS: Medium Resolution Imaging Spectrometer; MBO: Human bait landing catches outdoors ('Man-Biting Outdoors'); IVCC: Innovative Vector Control Consortium.

## Competing interests

The authors declare that they have no competing interests.

## Authors' contributions

SIH conceived the study and managed its design and implementation. MES and SIH wrote the first draft of the manuscript, MES assembled the occurrence data with assistance from CWK (also see Acknowledgements), CWK also digitised and edited all the expert opinion maps. WHT designed and maintained the databases and implemented the map figures. APP implemented the BRT scripts for predictive mapping. PWG processed the environmental and climatic data grids, with assistance from TVB. Experiments were derived by SIH and MES and implemented by MES. All authors participated in the interpretation of results and in the writing and editing of the manuscript. YR-P, SM and REH advised on bionomics and nomenclature issues, and provided additional comments and input to the manuscript.

## Supplementary Material

Additional file 1 **Expert opinion distribution maps for the nine DVS of the Americas**. Click here for file

Additional file 2 **Predictive species distribution maps for the nine DVS of the Americas**. Click here for file

Additional file 3 **Bionomics protocol**. Click here for file

Additional file 4 **Summary tables showing evaluation statistics for all mapping trials and final Boosted Regression Tree environmental and climatic variable selections for the final, optimal predictive maps**. Click here for file
