# Correction: The dominant *Anopheles *vectors of human malaria in the Americas: occurrence data, distribution maps and bionomic précis

**DOI:** 10.1186/1756-3305-4-210

**Published:** 2011-11-03

**Authors:** Marianne E Sinka, Yasmin Rubio-Palis, Sylvie Manguin, Anand P Patil, Will H Temperley, Peter W Gething, Thomas Van Boeckel, Caroline W Kabaria, Ralph E Harbach, Simon I Hay

**Affiliations:** 1 Spatial Ecology and Epidemiology Group, Tinbergen Building, Department of Zoology, University of Oxford, South Parks Road, Oxford OX1 3PS, UK; 2 BIOMED, Universidad de Carabobo, Apartado 2073, Maracay 2101-A, Venezuela; 3 Laboratorio de Ecología de Vectores, Dirección de Control de Vectores y Fauna Nociva, Ministerio del Poder Popular para la Salud, Maracay, Venezuela; 4 Institut de Recherche pour le Développement, Lab. d'Immuno-Physiopathologie Virale et Moleculaire, UMR-MD3/Univ. Montpellier I, Faculté de Pharmacie, 15, Ave Charles Flahault, 34093 Montpellier, France; 5 Biological Control and Spatial Ecology, Université Libre de Bruxelles CP160/12, Av FD Roosevelt 50, B1050, Brussels, Belgium; 6 Malaria Public Health and Epidemiology Group, Centre for Geographic Medicine, KEMRI - Univ. Oxford - Wellcome Trust Collaborative Programme, Kenyatta National Hospital Grounds, P.O. Box 43640-00100 Nairobi, Kenya; 7 Department of Entomology, The Natural History Museum, Cromwell Road, London, UK

## Correction

In our original publication detailing the distribution of the dominant vector species of malaria in the Americas (Sinka *et al. *[1]), both Figure one (The predicted distribution map of *An. darlingi*) and the *An. darlingi *map shown in Additional file two (The predicted distribution maps of the nine dominant vector species of the Americas) included points on the border between Costa Rica and Nicaragua. These are confirmed absence points and therefore should not have been included. These maps are intended to indicate locations only where the species presence has been confirmed. *Anopheles darlingi *has never been found or reported from Costa Rica or Nicaragua (as indicated in the Expert opinion map) despite numerous and comprehensive surveys in the area trying to locate it.

Copies of the corrected figure and the updated Additional file can be found in Figure 1 and Additional file 1 (in this publication) and are also available on the Malaria Atlas Project (MAP) website:

**Figure 1:**
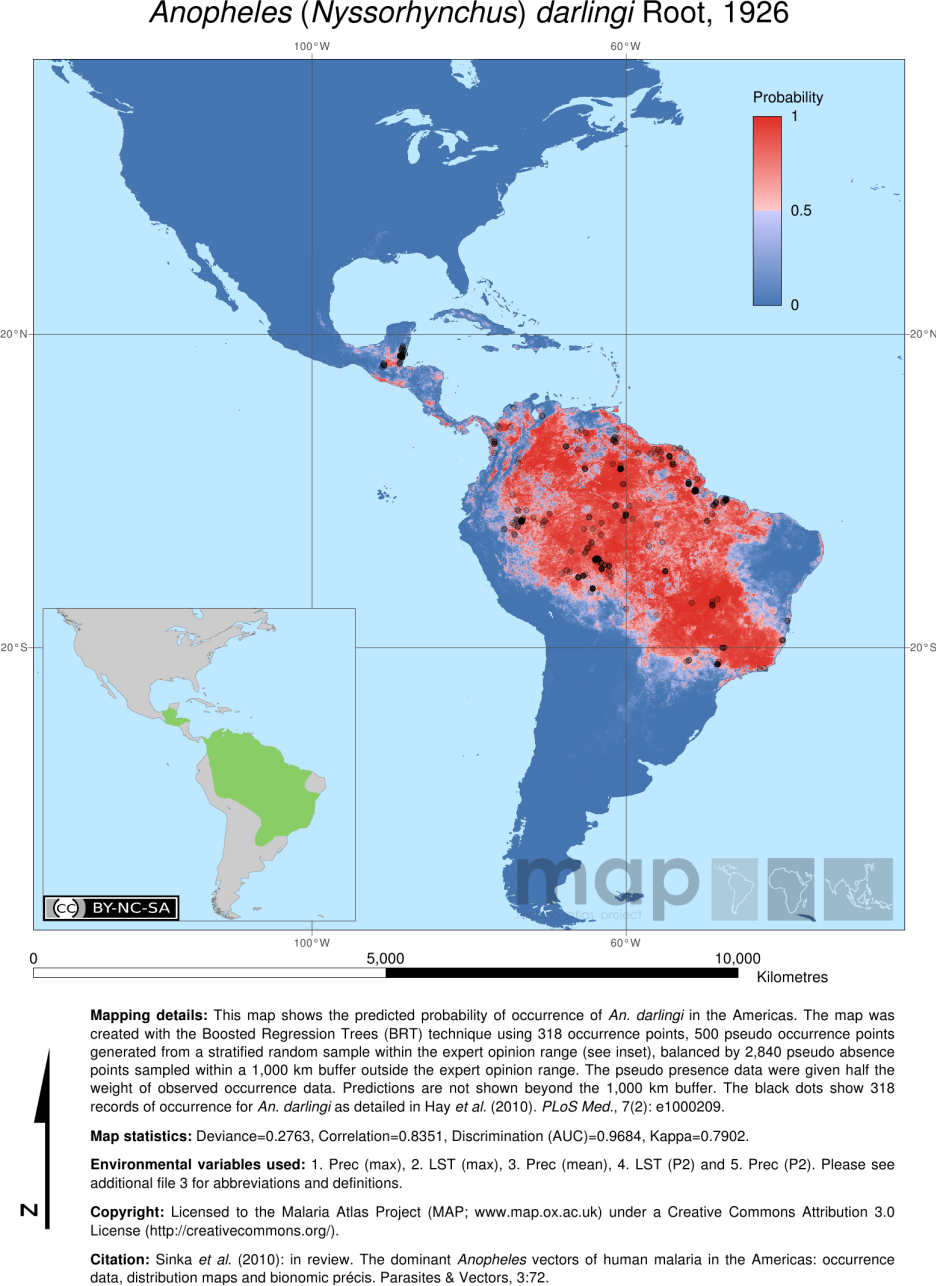
**Map details**: The predicted distribution of *An. darlingi *mapped using hybrid data (318 occurrence data plus 500 pseudo-presences weighted at half that of the occurrence data and randomly selected from within the Expert Opinion (EO) range). Pseudo-absences (2840) were generated at a ratio of 5:1 absence to presence points, taking into account 250 pseudo-presence points (500 at half weight), and were randomly selected from within the 1000 km buffer surrounding the EO (EO shown in the inset map). Predictions are not shown beyond the buffer boundary. The black dots show the 318 occurrence records for *An. darlingi*. **Map statistics**: Deviance = 0.2763, Correlation = 0.8351, Discrimination (AUC) = 0.9684, Kappa = 0.7902. **Environmental variables: **1. Prec (max), 2. LST (max), 3. Prec (mean), 4. LST (P2), 5. Prec (P2) (Please see Additional file four in the original publication for abbreviations and definitions). **Copyright**: Licensed to the Malaria Atlas Project [2] under a Creative Commons Attribution 3.0 License. **Citation: **Sinka *et al. *(2010) The dominant *Anopheles *vectors of human malaria in the Americas: occurrence data, distribution maps and bionomic précis, *Parasite and Vectors 2011, **4**:210*.

Figure One:

http://www.map.ox.ac.uk/media/PDF/Figure%201%20-%20An%20darlingi%20-%20corrected.png

Additional File Two (all species maps):

http://www.map.ox.ac.uk/media/PDF/Sinka%20et%20al_Additional%20file%202%20-%20final%20maps%20(FINAL).pdf

## Supplementary Material

Additional file 1 **Predictive species distribution maps for the nine DVS of the Americas**. Click here for file
