# Supplementary material for: The dominant Anopheles vectors of human malaria in the Americas: occurrence data, distribution maps and bionomic précis
Source: Parasit Vectors. 2010 Aug 16;3:72. doi: 10.1186/1756-3305-3-72 (PMC2936890; doi:10.1186/1756-3305-3-72)
Supplement: Additional file 2 — Predictive species distribution maps for the nine DVS of the Americas. [file 1756-3305-3-72-S2.PDF]

Anopheles (*Nyssorhynchus*) *albimanus* Wiedemann, 1820

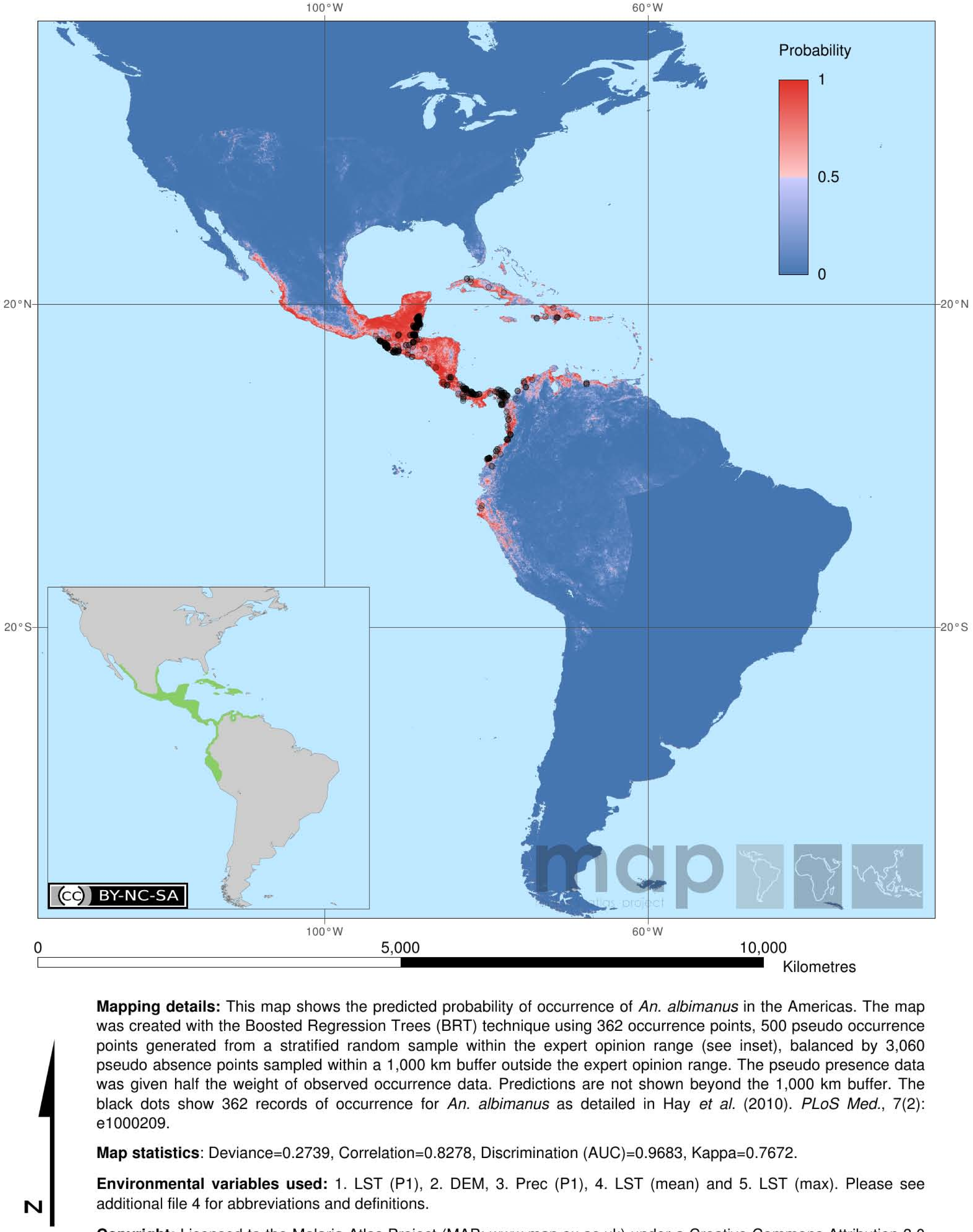

Mapping details: This map shows the predicted probability of occurrence of *An. albimanus* in the Americas. The map was created with the Boosted Regression Trees (BRT) technique using 362 occurrence points, 500 pseudo occurrence points generated from a stratified random sample within the expert opinion range (see inset), balanced by 3,060 pseudo absence points sampled within a 1,000 km buffer outside the expert opinion range. The pseudo presence data was given half the weight of observed occurrence data. Predictions are not shown beyond the 1,000 km buffer. The black dots show 362 records of occurrence for *An. albimanus* as detailed in Hay *et al.* (2010). *PLoS Med.*, 7(2): e1000209.

Map statistics: Deviance=0.2739, Correlation=0.8278, Discrimination (AUC)=0.9683, Kappa=0.7672.

Environmental variables used: 1. LST (P1), 2. DEM, 3. Prec (P1), 4. LST (mean) and 5. LST (max). Please see additional file 4 for abbreviations and definitions.

Copyright: Licensed to the Malaria Atlas Project (MAP; www.map.ox.ac.uk) under a Creative Commons Attribution 3.0 License (http://creativecommons.org/).

Citation: Sinka *et al.* (2010). The dominant *Anopheles* vectors of human malaria in the Americas: occurrence data, distribution maps and bionomic précis. *Parasites and Vectors* **3**:72.

Anopheles (*Nyssorhynchus*) *albitarsis* species complex

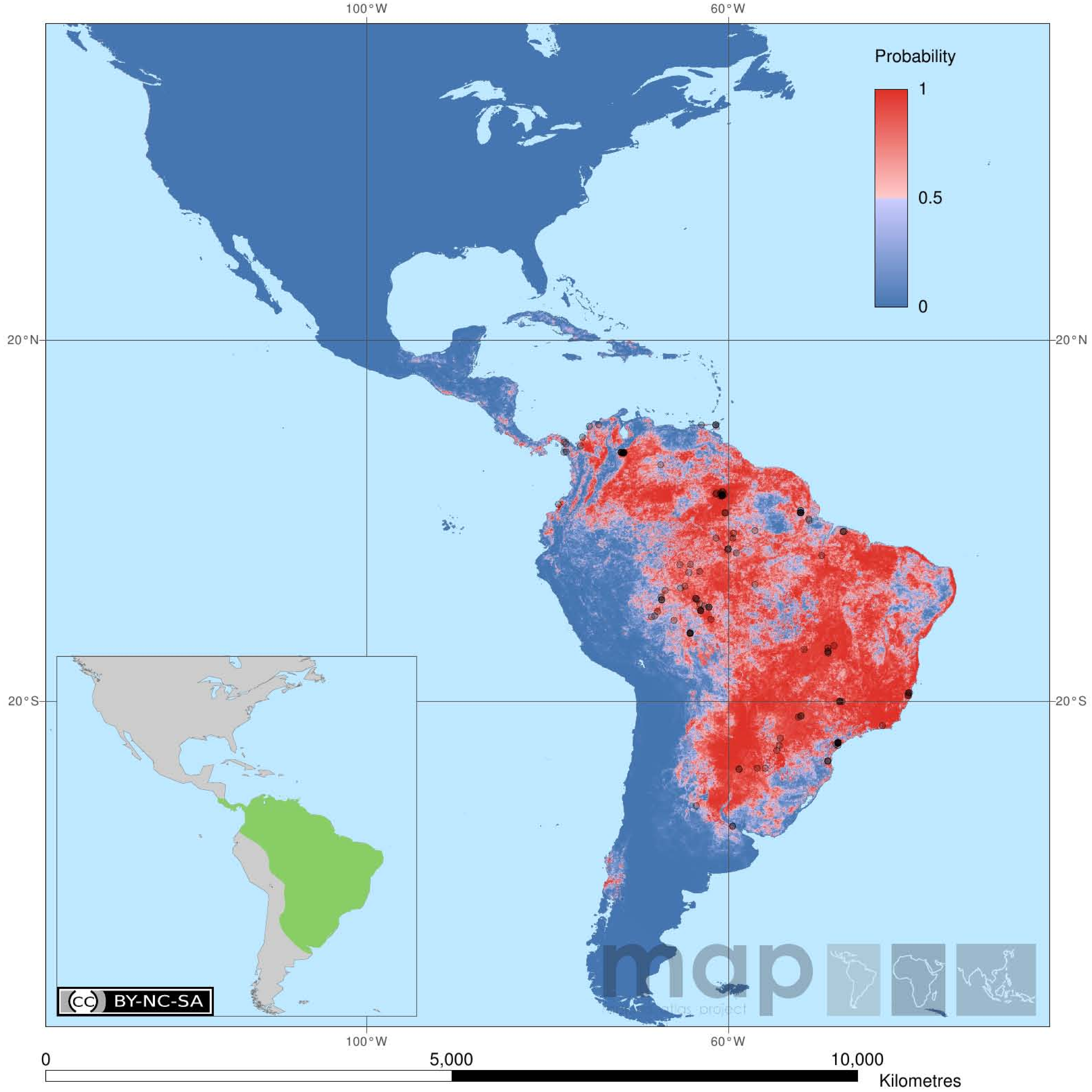

Mapping details: This map shows the predicted probability of occurrence of *An. albitarsis* in the Americas. The map was created with the Boosted Regression Trees (BRT) technique using 138 occurrence points, 500 pseudo occurrence points generated from a stratified random sample within the expert opinion range (see inset), balanced by 1,940 pseudo absence points sampled within a 1,000 km buffer outside the expert opinion range. The pseudo presence data was given half the weight of observed occurrence data. Predictions are not shown beyond the 1,000 km buffer. The black dots show 138 records of occurrence for *An. albitarsis* as detailed in Hay *et al.* (2010). *PLoS Med.*, 7(2): e1000209.

Map statistics: Deviance=0.2919, Correlation=0.8207, Discrimination (AUC)=0.9613, Kappa=0.7643.

Environmental variables used: 1. Prec (A1), 2. Prec (mean), 3. DEM, 4. LST (A1) and 5. NDVI (P1). Please see additional file 4 for abbreviations and definitions.

Copyright: Licensed to the Malaria Atlas Project (MAP; www.map.ox.ac.uk) under a Creative Commons Attribution 3.0 License (http://creativecommons.org/).

Citation: Sinka *et al.* (2010). The dominant *Anopheles* vectors of human malaria in the Americas: occurrence data, distribution maps and bionomic précis. *Parasites and Vectors* **3**:72.

Anopheles (*Nyssorhynchus*) *aquasalis* Curry, 1932

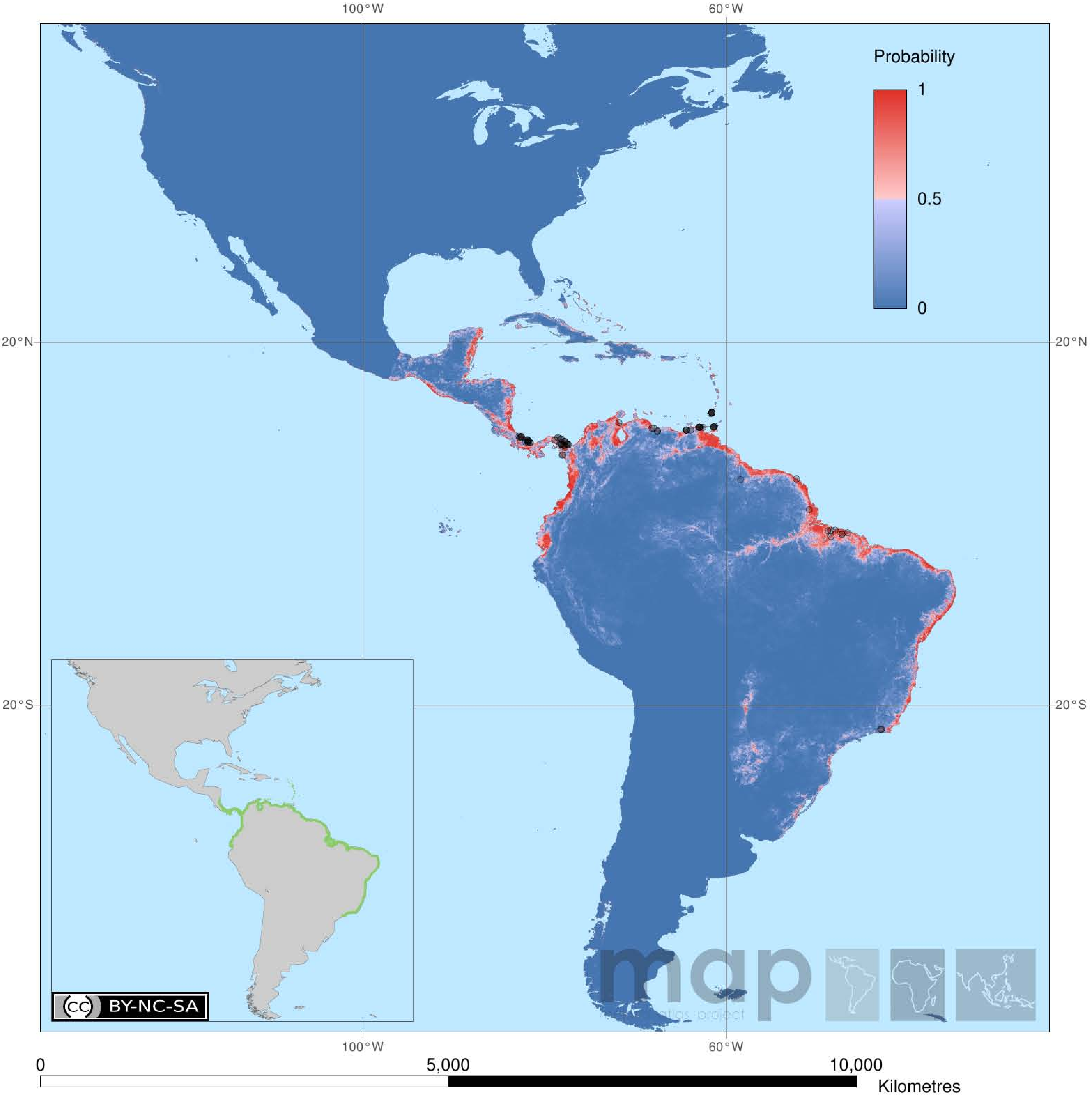

Mapping details: This map shows the predicted probability of occurrence of *An. aquasalis* in the Americas. The map was created with the Boosted Regression Trees (BRT) technique using 57 occurrence points, 500 pseudo occurrence points generated from a stratified random sample within the expert opinion range (see inset), balanced by 1,535 pseudo absence points sampled within a 1,000 km buffer outside the expert opinion range. The pseudo presence data was given half the weight of observed occurrence data. Predictions are not shown beyond the 1,000 km buffer. The black dots show 57 records of occurrence for *An. aquasalis* as detailed in Hay *et al.* (2010). *PLoS Med.*, 7(2): e1000209.

Map statistics: Deviance=0.3944, Correlation=0.7458, Discrimination (AUC)=0.9442, Kappa=0.6386.

Environmental variables used: 1. DEM, 2. Prec (P1), 3. Prec (A2), 4. LST (P1) and 5. LST (max). Please see additional file 4 for abbreviations and definitions.

Copyright: Licensed to the Malaria Atlas Project (MAP; www.map.ox.ac.uk) under a Creative Commons Attribution 3.0 License (http://creativecommons.org/).

Citation: Sinka *et al.* (2010). The dominant *Anopheles* vectors of human malaria in the Americas: occurrence data, distribution maps and bionomic précis. *Parasites and Vectors* **3**:72.

Anopheles (*Nyssorhynchus*) *darlingi* Root, 1926

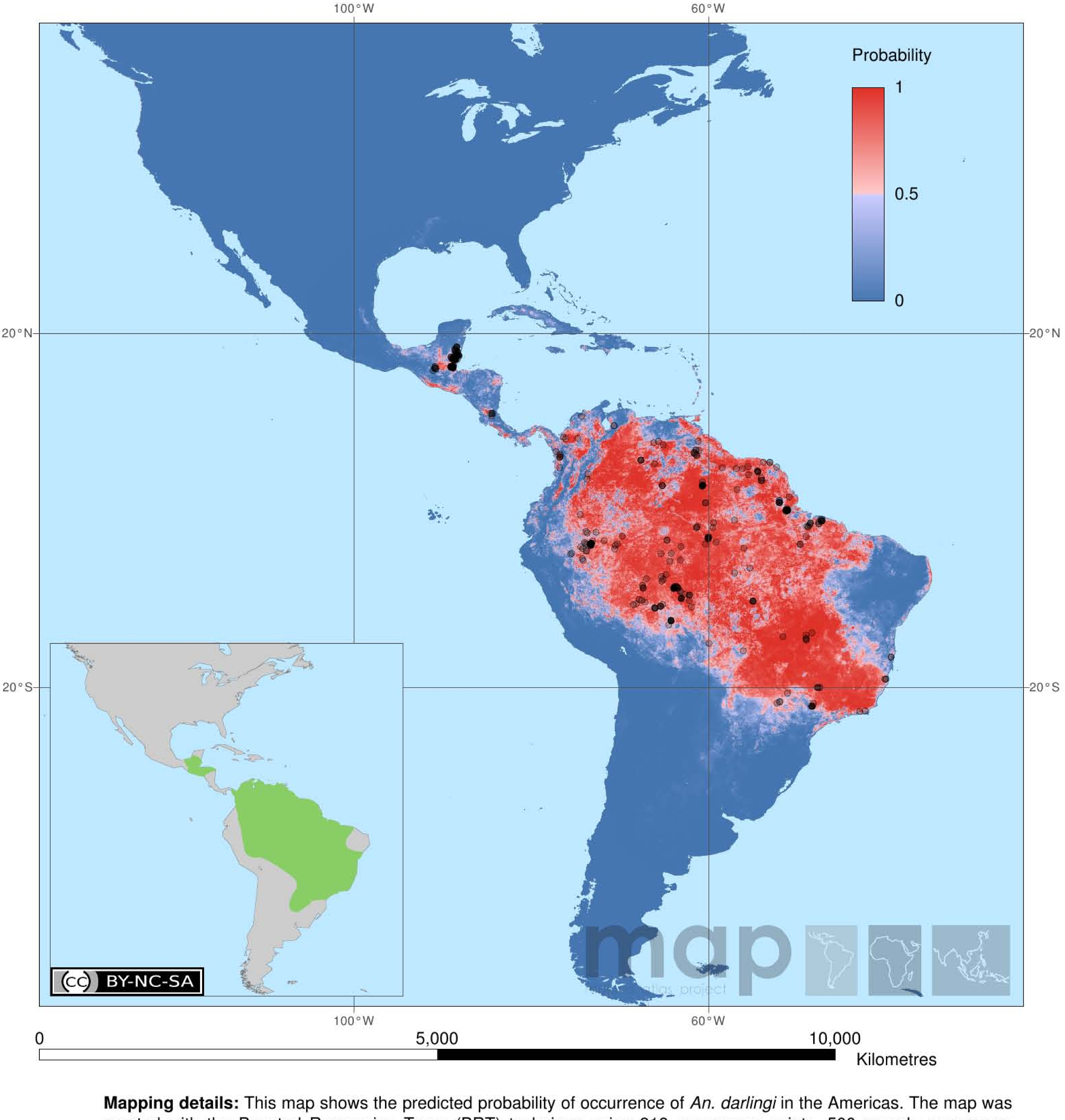

Mapping details: This map shows the predicted probability of occurrence of *An. darlingi* in the Americas. The map was created with the Boosted Regression Trees (BRT) technique using 318 occurrence points, 500 pseudo occurrence points generated from a stratified random sample within the expert opinion range (see inset), balanced by 2,840 pseudo absence points sampled within a 1,000 km buffer outside the expert opinion range. The pseudo presence data was given half the weight of observed occurrence data. Predictions are not shown beyond the 1,000 km buffer. The black dots show 318 records of occurrence for *An. darlingi* as detailed in Hay *et al.* (2010). *PLoS Med.*, 7(2): e1000209.

Map statistics: Deviance=0.2763, Correlation=0.8351, Discrimination (AUC)=0.9684, Kappa=0.7902.

Environmental variables used: 1. Prec (max), 2. LST (max), 3. Prec (mean), 4. LST (P2) and 5. Prec (P2). Please see additional file 4 for abbreviations and definitions.

Copyright: Licensed to the Malaria Atlas Project (MAP; www.map.ox.ac.uk) under a Creative Commons Attribution 3.0 License (http://creativecommons.org/).

Citation: Sinka *et al.* (2010). The dominant *Anopheles* vectors of human malaria in the Americas: occurrence data, distribution maps and bionomic précis. *Parasites and Vectors* **3**:72.

Anopheles (*Anopheles*) *freeborni* Aitken, 1939

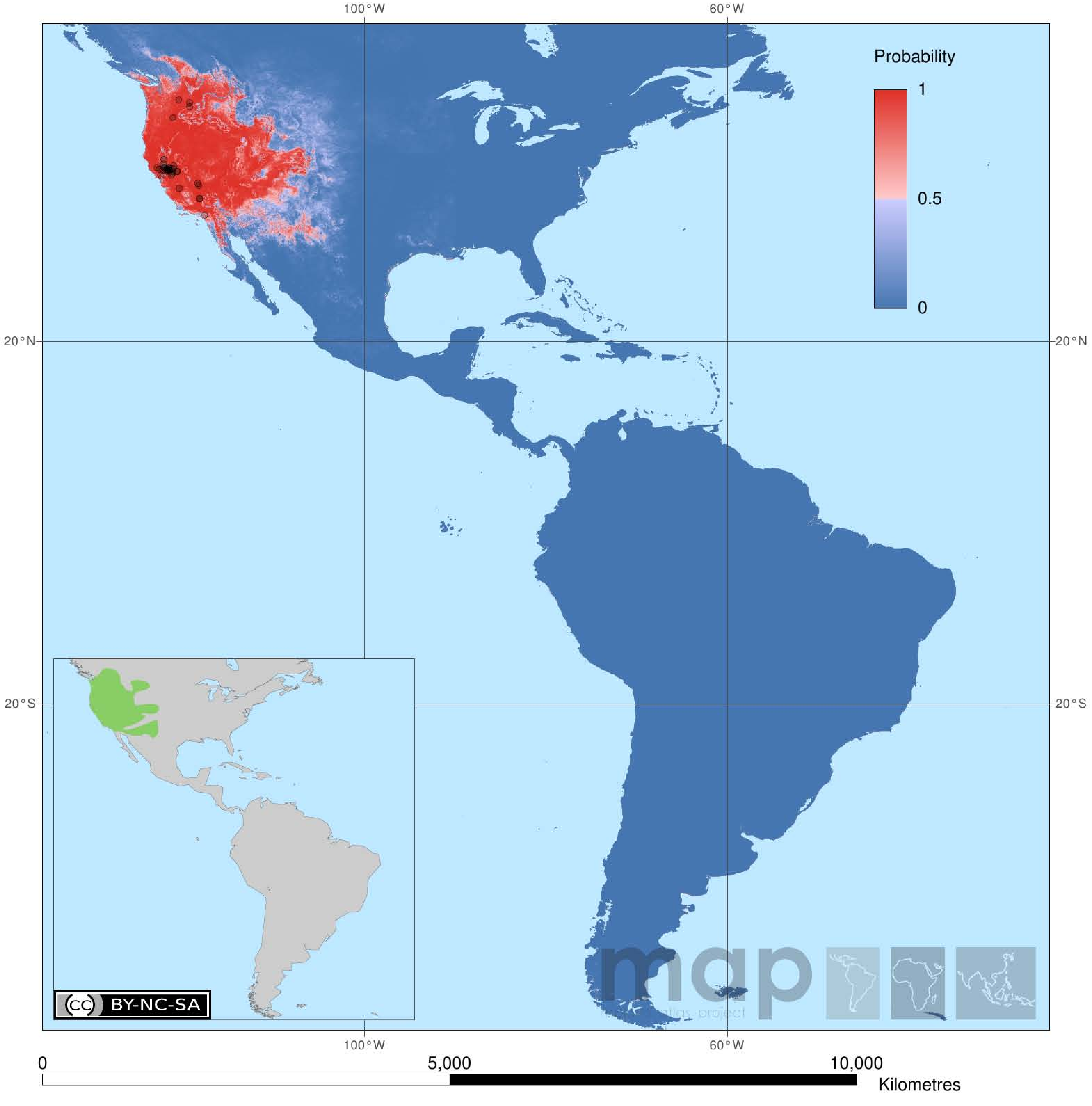

N

Mapping details: This map shows the predicted probability of occurrence of *An. freeborni* in the Americas. The map was created with the Boosted Regression Trees (BRT) technique using 37 occurrence points, 500 pseudo occurrence points generated from a stratified random sample within the expert opinion range (see inset), balanced by 1,435 pseudo absence points sampled within a 1,000 km buffer outside the expert opinion range. The pseudo presence data was given half the weight of observed occurrence data. Predictions are not shown beyond the 1,000 km buffer. The black dots show 37 records of occurrence for *An. freeborni* as detailed in Hay *et al.* (2010). *PLoS Med.*, 7(2): e1000209.

Map statistics: Deviance=0.2932, Correlation=0.8191, Discrimination (AUC)=0.9666, Kappa=0.7573.

Environmental variables used: 1. Prec (P1), 2. Prec (A1), 3. Prec (max), 4. DEM and 5. LST (A1). Please see additional file 4 for abbreviations and definitions.

Copyright: Licensed to the Malaria Atlas Project (MAP; www.map.ox.ac.uk) under a Creative Commons Attribution 3.0 License (http://creativecommons.org/).

Citation: Sinka *et al.* (2010). The dominant *Anopheles* vectors of human malaria in the Americas: occurrence data, distribution maps and bionomic précis. *Parasites and Vectors* **3**:72.

Anopheles (*Nyssorhynchus*) *marajoara* Galvão & Damasceno, 1942

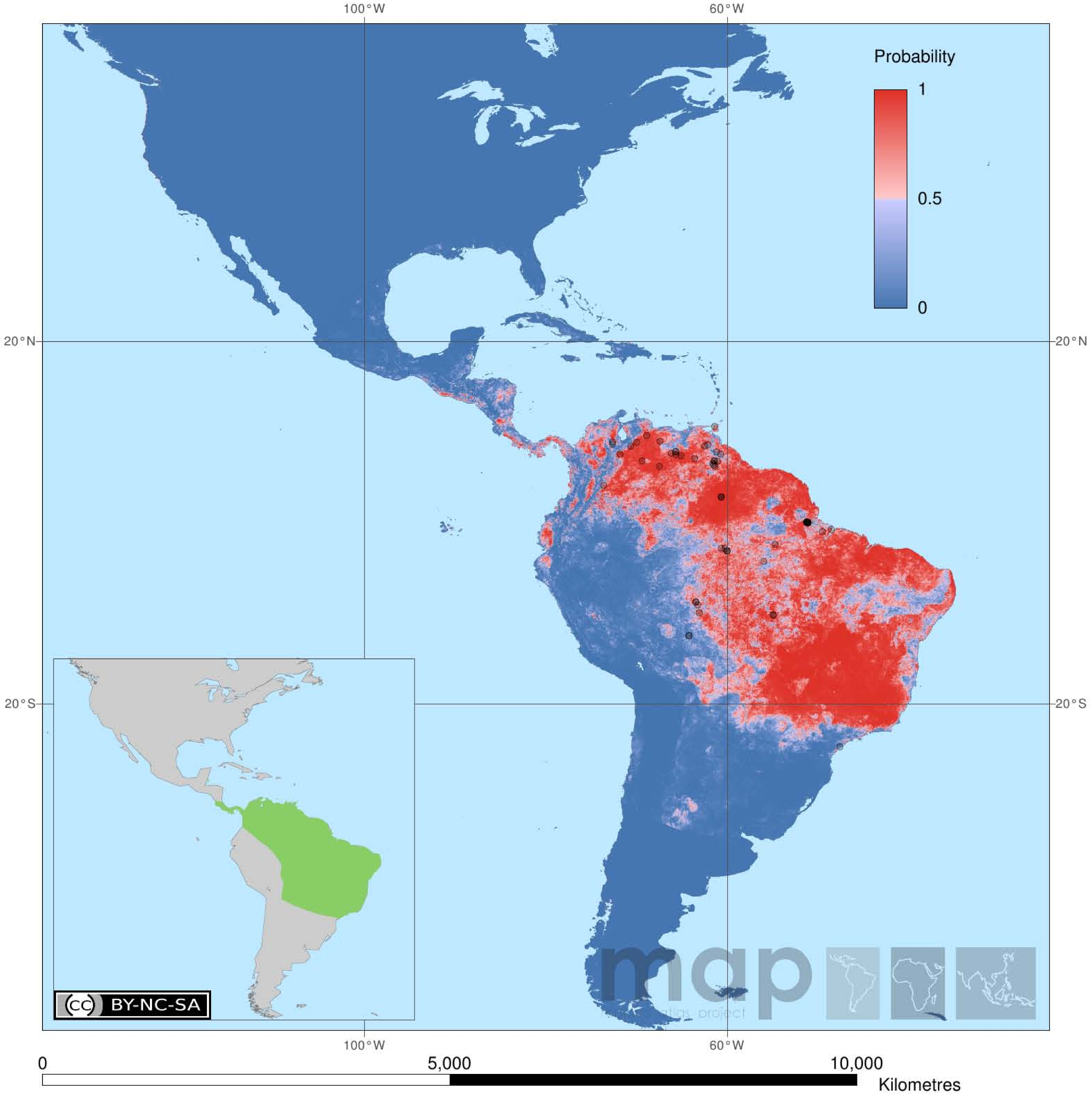

Mapping details: This map shows the predicted probability of occurrence of *An. marajoara* (formerly *An. albitarsis* sp. C) in the Americas. The map was created with the Boosted Regression Trees (BRT) technique using 56 occurrence points, 500 pseudo occurrence points generated from a stratified random sample within the expert opinion range (see inset), balanced by 1,530 pseudo absence points sampled within a 1,000 km buffer outside the expert opinion range. The pseudo presence data was given half the weight of observed occurrence data. Predictions are not shown beyond the 1,000 km buffer. The black dots show 56 records of occurrence for *An. marajoara* as detailed in Hay *et al.* (2010). *PLoS Med.*, 7(2): e1000209.

Map statistics: Deviance=0.3354, Correlation=0.7962, Discrimination (AUC)=0.9482, Kappa=0.7219.

Environmental variables used: 1. Prec (P2), 2. Prec (A1), 3. LST (P2), 4. NDVI (A1) and 5. Prec (min). Please see additional file 4 for abbreviations and definitions.

N

Copyright: Licensed to the Malaria Atlas Project (MAP; www.map.ox.ac.uk) under a Creative Commons Attribution 3.0 License (http://creativecommons.org/).

Citation: Sinka *et al.* (2010). The dominant *Anopheles* vectors of human malaria in the Americas: occurrence data, distribution maps and bionomic précis. *Parasites and Vectors* **3**:72.

Anopheles (*Nyssorhynchus*) *nuneztovari* species complex

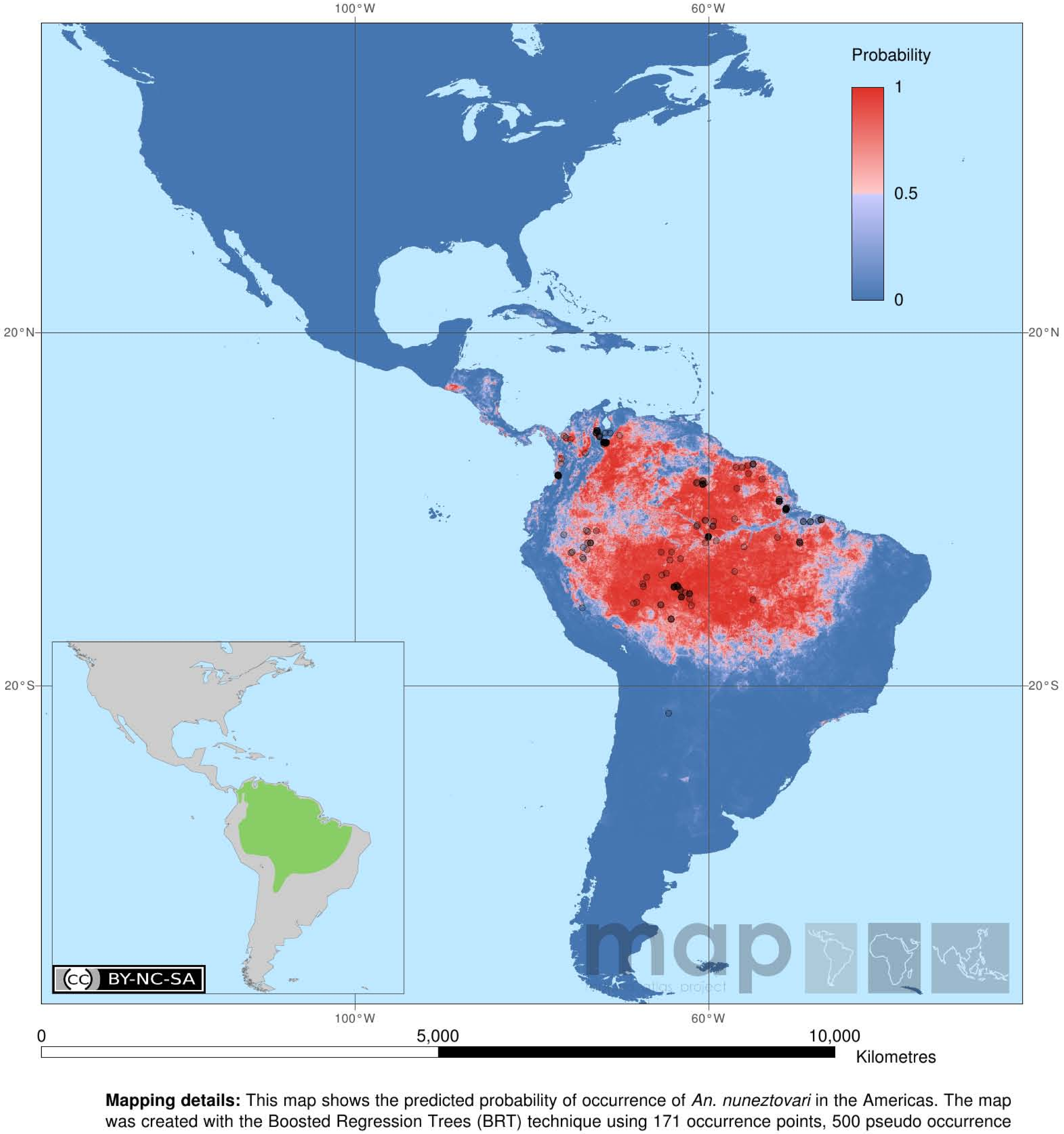

Mapping details: This map shows the predicted probability of occurrence of *An. nuneztovari* in the Americas. The map was created with the Boosted Regression Trees (BRT) technique using 171 occurrence points, 500 pseudo occurrence points generated from a stratified random sample within the expert opinion range (see inset), balanced by 2,105 pseudo absence points sampled within a 1,000 km buffer outside the expert opinion range. The pseudo presence data was given half the weight of observed occurrence data. Predictions are not shown beyond the 1,000 km buffer. The black dots show 171 records of occurrence for *An. nuneztovari* as detailed in Hay *et al.* (2010). *PLoS Med.*, 7(2): e1000209.

Map statistics: Deviance=0.3094, Correlation=0.8161, Discrimination (AUC)=0.9555, Kappa=0.7633.

Environmental variables used: 1. Prec (max), 2. Prec (mean), 3. LST (mean), 4. Prec (A1) and 5. LST (min). Please see additional file 4 for abbreviations and definitions.

Copyright: Licensed to the Malaria Atlas Project (MAP; www.map.ox.ac.uk) under a Creative Commons Attribution 3.0 License (http://creativecommons.org/).

Citation: Sinka *et al.* (2010). The dominant *Anopheles* vectors of human malaria in the Americas: occurrence data, distribution maps and bionomic précis. *Parasites and Vectors* **3**:72.

Anopheles (*Anopheles*) *pseudopunctipennis* species complex

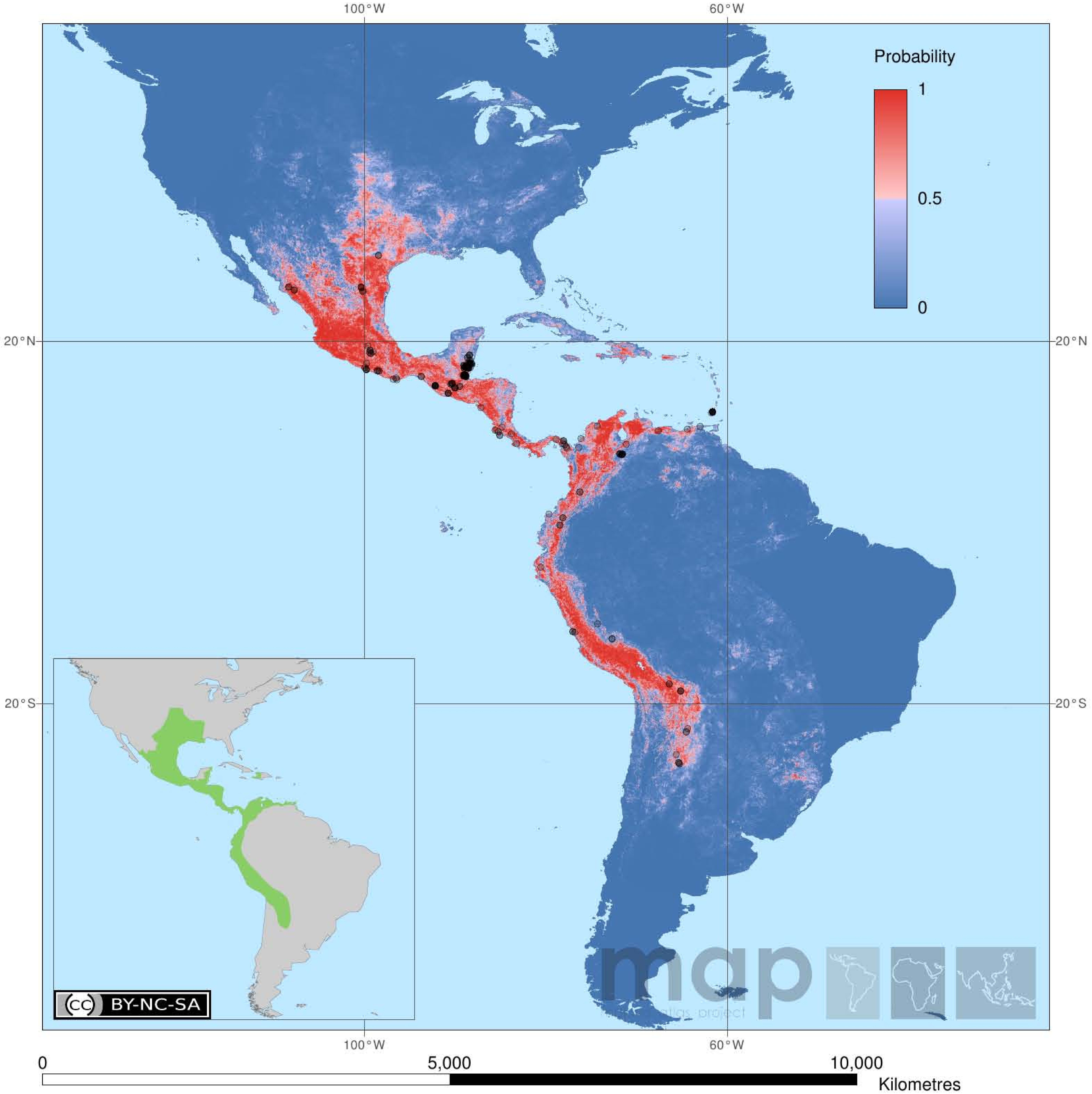

Mapping details: This map shows the predicted probability of occurrence of *An. pseudopunctipennis* in the Americas. The map was created with the Boosted Regression Trees (BRT) technique using 156 occurrence points, 500 pseudo occurrence points generated from a stratified random sample within the expert opinion range (see inset), balanced by 2,030 pseudo absence points sampled within a 1,000 km buffer outside the expert opinion range. The pseudo presence data was given half the weight of observed occurrence data. Predictions are not shown beyond the 1,000 km buffer. The black dots show 156 records of occurrence for *An. pseudopunctipennis* as detailed in Hay *et al.* (2010). *PLoS Med.*, 7(2): e1000209.

Map statistics: Deviance=0.3567, Correlation=0.7682, Discrimination (AUC)=0.9432, Kappa=0.6768.

Environmental variables used: 1. LST (P1), 2. DEM, 3. Prec (P1), 4. MIR (mean) and 5. MIR (P1). Please see additional file 4 for abbreviations and definitions.

Copyright: Licensed to the Malaria Atlas Project (MAP; www.map.ox.ac.uk) under a Creative Commons Attribution 3.0 License (http://creativecommons.org/).

Citation: Sinka *et al.* (2010). The dominant *Anopheles* vectors of human malaria in the Americas: occurrence data, distribution maps and bionomic précis. *Parasites and Vectors* **3**:72.

Anopheles (*Anopheles*) *quadrimaculatus* Say, 1824

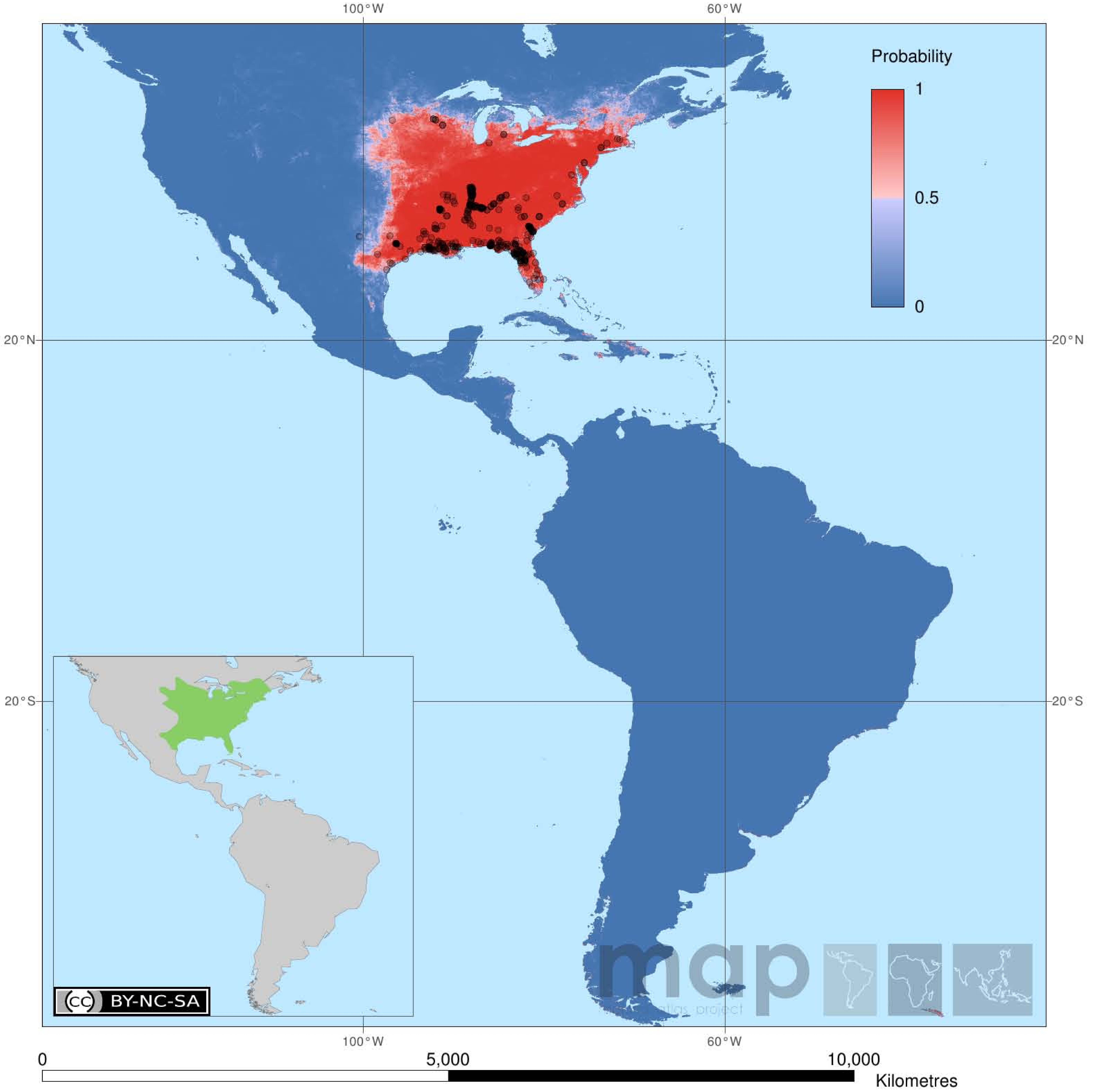

Mapping details: This map shows the predicted probability of occurrence of *An. quadrimaculatus* in the Americas. The map was created with the Boosted Regression Trees (BRT) technique using 379 occurrence points, 500 pseudo occurrence points generated from a stratified random sample within the expert opinion range (see inset), balanced by 3,145 pseudo absence points sampled within a 1,000 km buffer outside the expert opinion range. The pseudo presence data was given half the weight of observed occurrence data. Predictions are not shown beyond the 1,000 km buffer. The black dots show 379 records of occurrence for *An. quadrimaculatus* as detailed in Hay *et al.* (2010). *PLoS Med.*, 7 (2): e1000209.

Map statistics: Deviance=0.1237, Correlation=0.9271, Discrimination (AUC)=0.9904, Kappa=0.908.

Environmental variables used: 1. Prec (min), 2. Prec (mean), 3. Prec (P1), 4. LST (A1) and 5. DEM. Please see additional file 4 for abbreviations and definitions.

Copyright: Licensed to the Malaria Atlas Project (MAP; www.map.ox.ac.uk) under a Creative Commons Attribution 3.0 License (http://creativecommons.org/).

Citation: Sinka *et al.* (2010). The dominant *Anopheles* vectors of human malaria in the Americas: occurrence data, distribution maps and bionomic précis. *Parasites and Vectors* **3**:72.
