# Supplementary material for: The dominant Anopheles vectors of human malaria in the Americas: occurrence data, distribution maps and bionomic précis
Source: Parasit Vectors. 2010 Aug 16;3:72. doi: 10.1186/1756-3305-3-72 (PMC2936890; doi:10.1186/1756-3305-3-72)
Supplement: Additional file 3 — Bionomics protocol. [file 1756-3305-3-72-S3.DOC]

**Additional file 3:** Bionomics protocol.

**Each spread sheet refers to a single species. Only enter data for ‘wild’ mosquitoes (i.e. not lab colonies), to ensure we only incorporate field bionomics rather than laboratory colony behaviour.**

**Literature source**

1. ENLID – Enter the unique record of the PDF source examined.
2. Author – Enter the surname of the first author of the publication only.
3. Year – Enter the year of publication.
4. Bionomics info? – Enter from the drop down menu ‘Y’ if the source contains bionomics information for the species in question and ‘N’ if it does not. Enter ‘limited’ if the information includes, for example, sampling method which will indicate that a species has been captured on a certain host, but no choice was given to indicate biting preference.
5. Secondary or general Info – Note any additional secondary information given in the text that is relevant to the species’ bionomics, for example ‘This is a known zoophilic species’.

**Larval Site Characteristics (LSC) – characteristics are not exclusive, ‘Y’ can be entered into more than one column.**

1. (spacer column).
2. LSC – Light – Heliophilic – Enter ‘Y’ if larvae were found in sunlit larval sites.
3. LSC – Light – Heliophobic – Enter ‘Y’ if larvae were found in shaded larval sites.
4. LSC – Salinity – High (brackish) – Enter ‘Y’ if larvae were found in brackish larval sites.
5. LSC – Salinity – Low (fresh) – Enter ‘Y’ if larvae were found in fresh water larval sites.
6. LSC – Turbidity ( Clear) – Enter ‘Y’ if larvae were found in clear water larval sites.
7. LSC – Turbidity (Polluted) – Enter ‘Y’ if larvae were found in turbid and/or polluted larval sites.
8. LSC – Movement (Still or stagnant) – Enter ‘Y’ if larvae were found in larval sites with still or stagnant water.
9. LSC – Movement (Flowing) - Enter ‘Y’ if larvae were found in larval sites with moving or flowing water.
10. LSC – Vegetation (Higher plants, algae etc.) - Enter ‘Y’ if larvae were found in larval sites within vegetation, including higher plants, or algae.
11. LSC – Vegetation (No vegetation) - Enter ‘Y’ if larvae were found in larval sites with no associated vegetation.
12. LSC – Larval site notes – Enter any notes relevant to the larval site characteristics, for example: Species A was only found in association with filamentous algae.

**Large Natural Water Collections (LNWC) – sites are not exclusive, ‘Y’ can be entered in more than one column.**

1. (spacer column).
2. LNWC – Lagoons – Enter ‘Y’ if larvae were sampled from a lagoon.
3. LNWC – Lakes – Enter ‘Y’ if larvae were sampled from a lake.
4. LNWC – Marshes – Enter ‘Y’ if larvae were sampled from a marsh.
5. LNWC – Bogs – Enter ‘Y’ if larvae were sampled from a bog.
6. LNWC – Slow flowing rivers – Enter ‘Y’ if larvae were sampled from a slow flowing river NOTE: if the sampling was from river margins (i.e. in areas with no water flow) enter these in the next column: (X. ‘Other’) and make a note in the site notes.
7. LNWC – Other – Enter ‘Y’ if larvae were sampled from a LNWC site other than those given in columns S to W. Note the type of site in the LNWC site notes.
8. LNWC – site notes – Enter details of any LNWC sites that have been marked in column X (‘Other’). Note any information relevant to entries in columns S to W, for example, size or depth of the water body, if given.

**Large Man-Made Water Collections (LM-MWC) – sites are not exclusive, ‘Y’ can be entered in more than one column.**

1. (spacer column).
2. LM-MWC – Borrow pits – Enter ‘Y’ if larvae were sampled from a large borrow pit.

AB. LM-MWC – Rice fields – Enter ‘Y’ if larvae were sampled from a rice field.

AC. LM-MWC – Fish ponds – Enter ‘Y’ if larvae were sampled from a fish pond.

AD. LM-MWC – Irrigation channels – Enter ‘Y’ if larvae were sampled from irrigation channels.

AE. LM-MWC – Other – Enter ‘Y’ if larvae were sampled from a LM-MWC site other than those given in columns AA to AD. Note the type of site in the LM-MWC site notes.

AF. LM-MWC – site notes – Enter details of any LM-MWC sites that have been marked in column AE (‘Other’). Note any information relevant to entries in columns AA to AD, for example, size or depth of the water body, if given.

**Small Natural Water Collections (SNWC) – sites are not exclusive, ‘Y’ can be entered in more than one column.**

AG. (spacer column).

AH. SNWC – Small streams – Enter ‘Y’ if larvae were sampled from a small stream.

AI. SNWC – Seepage springs – Enter ‘Y’ if larvae were sampled from a seepage spring.

AJ. SNWC – Pools – Enter ‘Y’ if larvae were sampled from a small pool.

AK. SNWC – Wells – Enter ‘Y’ if larvae were sampled from a well.

AL. SNWC – Depressions in the ground – Enter ‘Y’ if larvae were sampled from depressions in the ground.

AM.SNWC – Other – Enter ‘Y’ if larvae were sampled from a SNWC site other than those given in columns AH to AL. Note the type of site in the SNWC site notes.

AN. SNWC – site notes – Enter details of any SNWC sites that have been marked in column AM (‘Other’). Note any information relevant to entries in columns AH to AL, for example, size or depth of the water body, if given.

**Small Man-Made Water Collections (SM-MWC) – sites are not exclusive, ‘Y’ can be entered in more than one column.**

AO. (spacer column).

AP. SM-MWC – Overflow water – Enter ‘Y’ if larvae were sampled from overflow water.

AQ. SM-MWC – Irrigation ditches – Enter ‘Y’ if larvae were sampled from irrigation ditches.

AR. SM-MWC – Borrow pits – Enter ‘Y’ if larvae were sampled from a small borrow pits.

AS. SM-MWC – Wheel ruts – Enter ‘Y’ if larvae were sampled from wheel ruts.

AT. SM-MWC – Hoof prints – Enter ‘Y’ if larvae were sampled from hoof prints.

AU. SM-MWC – Puddles near rice fields – Enter ‘Y’ if larvae were sampled from puddles near rice fields.

AV. SM-MWC – Other – Enter ‘Y’ if larvae were sampled from a SM-MWC site other than those given in columns AP to AU. Note the type of site in the SM-MWC site notes.

AW.SM-MWC – site notes – Enter details of any SM-MWC sites that have been marked in column AV (‘Other’). Note any information relevant to entries in columns AP to AU, for example, size or depth of the water body, if given.

**Artificial Sites (AS) – sites are not exclusive, ‘Y’ can be entered in more than one column.**

AX. (spacer column).

AY. AS – Empty cans, shells, etc. – Enter ‘Y’ if larvae were sampled from any small artificial containers.

AZ. AS – site notes – Note any information relevant to entries in column AS, for example, the type of container the larvae were found in.

**Adult – General (AG) – ‘No preference’ is indicated with ‘Y’ in both columns**

BA. (spacer column).

BB. AG – Exophilic – Enter ‘Y’ if article reports general exophilic behaviour of the adult, where it cannot be easily categorised as resting or biting exophilly, or where exophilic resting behaviour cannot be categorised as pre or post feeding.

BC. AG – Endophilic – Enter ‘Y’ if article reports general endophilic behaviour of the adult, where it cannot be easily categorised as resting or biting enophilly, or where endophilic resting behaviour cannot be categorised as pre or post feeding.

BD. AG Exo/Endophilic notes – Note the reasoning for entering data in columns BB or BC, for example: the article reports ambiguous results that cannot be assigned to biting or resting habits: ‘More mosquitoes were collected indoors than outdoors’.

**Adult Biting Activity (ABA) – ‘Y’ can be entered in more than one column if ‘no preference’ is reported**

BE. (spacer column).

BF. ABA - Feeding habit – Anthropophilic – Enter ‘Y’ from the drop down menu if there is clear evidence of a preference for human hosts or ‘?’ if the mosquitoes were attracted to or biting human hosts, but no preference was indicated, for example where sampling was only conducted using man-biting techniques. Note any relevant information in column BN (‘Biting notes’), for example: ‘of the 770 mosquitoes that had fed on mammals, only 2 had fed on humans’.

BG. ABA - Feeding habit – Zoophilic – Enter ‘Y’ from the drop down menu if there is clear evidence of a preference for animal hosts or ‘?’ if the mosquitoes were attracted to or biting animal hosts, but no preference was indicated, for example where sampling was only conducted using animal baited traps. Note any relevant information in column BN (‘Biting notes’), for example: ‘a large proportion fed on mammals, including cow, horse and deer’.

BH. ABA - Biting habit – Exophagic – Enter ‘Y’ from the drop down menu if there is clear evidence of a preference for biting outdoors or ‘?’ if the mosquitoes were sampled biting outdoors, but no preference was indicated, for example, mosquitoes were only collected from outdoor sites or biting outdoors. Note any relevant information in column BN (‘Biting notes’), for example: proportions found biting indoors and outdoors.

BI. ABA - Biting habit – Endophagic – Enter ‘Y’ from the drop down menu if there is clear evidence of a preference for biting indoors or ‘?’ if the mosquitoes were sampled biting indoors, but no preference was indicated, for example, mosquitoes were only collected resting or biting indoors. Note any relevant information in column BN (‘Biting notes’), for example: proportions found biting indoors and outdoors.

BJ. ABA - Biting time – Day – Enter ‘Y’ if the biting peak occurred during the day. There can be more than one biting peak so entries should be made in all relevant categories. Make a note of the time of the biting peak(s) in column BN (‘Biting notes’), plus any other information relevant to the biting habit or period.

BK. ABA - Biting time – Dusk – Enter ‘Y’ if the biting peak occurred at dusk. There can be more than one biting peak so entries should be made in all relevant categories. Make a note of the time of the biting peak in column BN (‘Biting notes’), plus any other information relevant to the biting habit or period.

BL. ABA - Biting time – Night – Enter ‘Y’ if the biting peak occurred during the night. There can be more than one biting peak so entries should be made in all relevant categories, plus any other information relevant to the biting habit or period.

BM. ABA - Biting time – Dawn – Enter ‘Y’ if the biting peak occurred at dawn. There can be more than one biting peak so entries should be made in all relevant categories, plus any other information relevant to the biting habit or period.

BN. ABA Biting notes – Note any relevant information related to columns BF to BM.

**Adult Resting (AR) – ‘Y’ can be entered in more than one column if ‘no preference’ is reported**

BO. (spacer column).

BP. AR - Pre-feeding resting – Exophilic – Enter ‘Y’ if un-engorged resting mosquitoes were collected outdoors.

BQ. AR - Pre-feeding resting – Endophilic – Enter ‘Y’ if un-engorged resting mosquitoes were collected indoors.

BR. AR - Post-feeding resting – Exophilic – Enter ‘Y’ if engorged resting mosquitoes were collected outdoors.

BS. AR - Post-feeding resting – Endophilic – Enter ‘Y’ if engorged resting mosquitoes were collected indoors.

BT. AR resting notes – note any information relevant to the resting habits of the mosquito.

**Behaviour Relevant Control (BRC)**

BU. (spacer column).

BV. BRC – Enter ‘Y’ if the article indicates that observed or measured behaviour is altered as a result of vector control measures in place in the survey area, for example, if mosquitoes exhibit greater outdoor resting or biting after IRS control measures, compared to areas where no control has been applied.

BW. BRC notes – Note information relevant to data entered in column BV.

**Flight range**

BX. (spacer column).

BY. Flight range (km) – Daily – Enter the number of km reported for the daily flight range.

BZ. Flight range (km) – lifetime – enter the number of km reported for the lifetime flight range.

CA. Flight notes – Note any information relevant to columns BY and BZ.

**General information**

CB. (spacer column).

CC. General Notes – note the country and state in which the sampling was done, plus any other information relevant that is not included in other note fields.

CD. Useful refs. – List any references given in the article that may have useful bionomics information (usually in the Introduction or Discussion) with a note to what characteristic they may refer to, for example: Downs et al (1943), Senior White (1953) - biting time.
