# Supplementary material for: The dominant Anopheles vectors of human malaria in the Americas: occurrence data, distribution maps and bionomic précis
Source: Parasit Vectors. 2010 Aug 16;3:72. doi: 10.1186/1756-3305-3-72 (PMC2936890; doi:10.1186/1756-3305-3-72)
Supplement: Additional file 4 — Summary tables showing evaluation statistics for all mapping trials and final Boosted Regression Tree environmental and climatic variable selections for the final, optimal predictive maps. [file 1756-3305-3-72-S4.DOC]

**Additional file 4:** Summary tables showing evaluation statistics for all mapping trials and final BRT environmental and climatic variable selection for final, optimal predictive maps.

**Table 4.1**. Environmental and climatic variables grids available to the BRT species mapping listing the abbreviations used in the mapping figures.

| **File name** | **Abbreviation** | **Description** |
| --- | --- | --- |
| Wd0103a0 | MIR (mean) | Middle Infrared (MIR) - mean |
| wd0103a1 | MIR (A1) | Middle Infrared (MIR) - amplitude of the annual cycle |
| wd0103a2 | MIR (A2) | Middle Infrared (MIR) - amplitude of the bi-annual cycle |
| wd0103p1 | MIR (P1) | Middle Infrared (MIR) - phase of the annual cycle |
| wd0103p2 | MIR (P2) | Middle Infrared (MIR) - phase of the bi-annual cycle |
| wd0103mn | MIR (min) | Middle Infrared (MIR) - minimum |
| wd0103mx | MIR (max) | Middle Infrared (MIR) - maximum |
| wd0107a0 | LST (mean) | Land Surface Temperature (LST) - mean |
| wd0107a1 | LST (A1) | Land Surface Temperature (LST) - amplitude of the annual cycle |
| wd0107a2 | LST (A2) | Land Surface Temperature (LST) - amplitude of the bi-annual cycle |
| wd0107p1 | LST (P1) | Land Surface Temperature (LST) - phase of the annual cycle |
| wd0107p2 | LST (P2) | Land Surface Temperature (LST) - phase of the bi-annual cycle |
| wd0107mn | LST (min) | Land Surface Temperature (LST) - minimum |
| wd0107mx | LST (max) | Land Surface Temperature (LST) - maximum |
| wd0114a0 | NDVI (mean) | Normalized Difference Vegetation Index - mean |
| wd0114a1 | NDVI (A1) | Normalized Difference Vegetation Index - amplitude of the annual cycle |
| wd0114a2 | NDVI (A2) | Normalized Difference Vegetation Index - amplitude of the bi-annual cycle |
| wd0114p1 | NDVI (P1) | Normalized Difference Vegetation Index - phase of the annual cycle |
| wd0114p2 | NDVI (P2) | Normalized Difference Vegetation Index - phase of the bi-annual cycle |
| wd0114mn | NDVI (min) | Normalized Difference Vegetation Index - minimum |
| wd0114mx | NDVI (max) | Normalized Difference Vegetation Index - maximum |

**Table 4.1 (cont)**. Environmental and climatic variables grids available to the BRT species mapping listing the abbreviations used in the mapping figures.

| **File name** | **Abbreviation** | **Description** |
| --- | --- | --- |
| mod_dem | DEM | Digital Elevation Model (DEM) |
| prec57a0 | Prec (mean) | Precipitation - mean |
| prec57a1 | Prec (A1) | Precipitation - amplitude of the annual cycle |
| prec57a2 | Prec (A2) | Precipitation - amplitude of the bi-annual cycle |
| prec57mn | Prec (min) | Precipitation - minimum |
| prec57mx | Prec (max) | Precipitation - maximum |
| prec57p1 | Prec (P1) | Precipitation - phase of the annual cycle |
| prec57p2 | Prec (P2) | Precipitation - phase of the bi-annual cycle |
| globcover5k | GLOB-[ch. no.] | See table 3.2 |
| gc5k_dry | GLOB (dry) | Globcover – dry land cover classes [140, 150, 200] – see table 3.2 |
| gc5k_flo | GLOB (flood) | Globcover – flooded land cover classes [160, 170, 180] – see table 3.2 |
| gc5k_frs | GLOB (forest) | Globcover – forested land cover classes [40, 50, 60, 90, 100] – see table 3.2 |

**Table 4.2.** Globcover channels (land cover classes) available to the BRT species mapping (Channels 210: water bodies; 220: Permanent snow and ice and 230: no data were not included in the modelling).

| **Channel** | **Description** |
| --- | --- |
| 11 | Post-flooding or irrigated croplands (or aquatic) |
| 14 | Rainfed croplands |
| 20 | Mosaic cropland (50-70%)/vegetation (grassland/shrubland/forest) (20-50%) |
| 30 | Mosaic vegetation (grassland/shrubland/forest) (50-70%)/cropland (20-50%) |
| 40 | Closed to open (>15%) broadleaved evergreen or semi-deciduous forest (>5m) |
| 50 | Closed (>40%) broadleaved deciduous forest (>5m) |
| 60 | Open (15-40%) broadleaved deciduous forest/woodland (>5m) |
| 70 | Closed (>40%) needleleaved evergreen forest (>5m) |
| 90 | Open (15-40%) needleleaved deciduous or evergreen forest (>5m) |
| 100 | Closed to open (>15%) mixed broadleaved and needleleaved forest (>5m) |
| 110 | Mosaic forest or shrubland (50-70%) / grassland (20-50%) |
| 120 | Mosaic grassland (50-70%) / forest or shrubland (20-50%) |
| 130 | Closed to open (>15%) (broadleaved or needleleaved, evergreen or deciduous) shrubland (<5m) |
| 140 | Closed to open (>15%) herbaceous vegetation (grassland, savannas or lichens/mosses) |
| 150 | Sparse (<15%) vegetation |
| 160 | Closed to open (>15%) broadleaved forest regularly flooded (semi-permanently or temporarily) - Fresh or brackish water |
| 170 | Closed (>40%) broadleaved forest or shrubland permanently flooded - Saline or brackish water |
| 180 | Closed to open (>15%) grassland or woody vegetation on regularly flooded or waterlogged soil - Fresh, brackish or saline water |
| 190 | Artificial surfaces and associated areas (Urban areas >50%) |
| 200 | Bare areas |

**Table 4.3**: Evaluation metrics of mapping trials of data only maps (‘data’); expert opinion maps where 500 pseudo-presences were generated randomly within the EO range (‘EO’); and a combination of data and 500 pseudo-presences generated within the EO range, but given a weight rating of half the true data (‘hybrid’). All maps were run using a 1500 km buffer against 500 pseudo-absences using 5 x 5 km resolution.

|  |  | Metrics | | | |
| --- | --- | --- | --- | --- | --- |
| Species (no. of presence data) | Trial | Deviance  (0-1) | Correlation  (0-1) | Discrimination (AUC)  (0-1) | Kappa (κ)  (-1 to 1) |
| *An. albimanus* (362) | **data** | 0.2386 | 0.9355 | 0.9882 | 0.9187 |
|  | **EO** | 0.4342 | 0.8647 | 0.9687 | 0.8460 |
|  | **hybrid** | 0.1827 | 0.8919 | 0.9789 | 0.8661 |
| *An. albitarsis* (138) | **data** | 0.2596 | 0.8910 | 0.9845 | 0.8612 |
|  | **EO** | 0.5630 | 0.8245 | 0.9542 | 0.7820 |
|  | **hybrid** | 0.2885 | 0.8307 | 0.9572 | 0.7918 |
| *An. aquasalis* (57) | **data** | 0.3008 | 0.7569 | 0.9427 | 0.6741 |
|  | **EO** | 0.5938 | 0.8103 | 0.9444 | 0.7780 |
|  | **hybrid** | 0.3284 | 0.7970 | 0.9370 | 0.7566 |
| *An. darlingi* (318) | **data** | 0.3112 | 0.8995 | 0.9838 | 0.8651 |
|  | **EO** | 0.4993 | 0.8351 | 0.9633 | 0.7900 |
|  | **hybrid** | 0.2045 | 0.8760 | 0.9727 | 0.8470 |
| *An. freeborni* (37) | **data** | 0.0554 | 0.9398 | 0.9985 | 0.9183 |
|  | **EO** | 0.3484 | 0.8977 | 0.9775 | 0.8700 |
|  | **hybrid** | 0.2168 | 0.8632 | 0.9709 | 0.8222 |

**Table 4.3:** (cont.) Evaluation metrics of mapping trials of data only maps (‘data’); expert opinion maps where 500 pseudo-presences were generated randomly within the EO range (‘EO’); and a combination of data and 500 pseudo-presences generated within the EO range, but given a weight rating of half the true data. All maps were run using a 1500 km buffer against 500 pseudo-absences using 5 x 5 km resolution.

|  |  | Metrics | | | |
| --- | --- | --- | --- | --- | --- |
| Species | Buffer size (km) | Deviance  (0-1) | Correlation  (0-1) | Discrimination (AUC)  (0-1) | Kappa (κ)  (-1 to 1) |
| *An. marajoara* (56) | **data** | 0.2216 | 0.8161 | 0.9795 | 0.7458 |
|  | **EO** | 0.5116 | 0.8491 | 0.9605 | 0.8180 |
|  | **hybrid** | 0.2609 | 0.8343 | 0.9604 | 0.7831 |
| *An. nuneztovari* (171) | **data** | 0.3593 | 0.8577 | 0.9724 | 0.8292 |
|  | **EO** | 0.5392 | 0.8304 | 0.9530 | 0.7980 |
|  | **hybrid** | 0.2825 | 0.8318 | 0.9549 | 0.7996 |
| *An. pseudopunctipennis* (156) | **data** | 0.3081 | 0.8819 | 0.9765 | 0.8517 |
|  | **EO** | 0.6337 | 0.7844 | 0.9398 | 0.7380 |
|  | **hybrid** | 0.2727 | 0.8310 | 0.9548 | 0.7765 |
| *An. quadrimaculatus* (379) | **data** | 0.0844 | 0.9760 | 0.9990 | 0.9675 |
|  | **EO** | 0.2537 | 0.9241 | 0.9907 | 0.8960 |
|  | **hybrid** | 0.1209 | 0.9221 | 0.9916 | 0.8945 |

**Table 4.4**: Evaluation statistics for a range of buffer sizes, using 500 pseudo-absences at 5 x 5 km resolution.

|  |  | Metrics | | | |
| --- | --- | --- | --- | --- | --- |
| Species (no. of presence data) | Buffer size (km) | Deviance  (0-1) | Correlation  (0-1) | Discrimination (AUC)  (0-1) | Kappa (κ)  (-1 to 1) |
| *An. albimanus* (362) | **100** | 0.4325 | 0.8312 | 0.0052 | 0.8067 |
|  | **500** | 0.3065 | 0.8849 | 0.9816 | 0.8627 |
|  | **1000** | 0.2369 | 0.9127 | 0.9889 | 0.8893 |
|  | **1500** | 0.2357 | 0.9208 | 0.9884 | 0.9051 |
| *An. albitarsis* (138) | **100** | 0.2078 | 0.8665 | 0.9768 | 0.8300 |
|  | **500** | 0.2315 | 0.8423 | 0.9766 | 0.8084 |
|  | **1000** | 0.1776 | 0.8892 | 0.9837 | 0.8685 |
|  | **1500** | 0.2201 | 0.8601 | 0.9734 | 0.8154 |
| *An. aquasalis* (57) | **100** | 0.2313 | 0.6376 | 0.9322 | 0.5444 |
|  | **500** | 0.2287 | 0.6552 | 0.9412 | 0.5795 |
|  | **1000** | 0.2168 | 0.6685 | 0.9402 | 0.5357 |
|  | **1500** | 0.2056 | 0.6975 | 0.9418 | 0.5921 |
| *An. darlingi* (318) | **100** | 0.4353 | 0.8166 | 0.9605 | 0.7638 |
|  | **500** | 0.3386 | 0.8561 | 0.9760 | 0.8221 |
|  | **1000** | 0.2470 | 0.8987 | 0.9869 | 0.8825 |
|  | **1500** | 0.2591 | 0.8951 | 0.9857 | 0.8694 |
| *An. freeborni* (37) | **100** | 0.0510 | 0.9189 | 0.9962 | 0.9018 |
|  | **500** | 0.0524 | 0.9010 | 0.9978 | 0.8598 |
|  | **1000** | 0.0498 | 0.9200 | 0.9985 | 0.8824 |
|  | **1500** | 0.0476 | 0.9094 | 0.9990 | 0.8892 |

**Table 4.4:** (cont.) Evaluation statistics for a range of buffer sizes, using 500 pseudo-absences at 5 x 5 km resolution.

|  |  | Metrics | | | |
| --- | --- | --- | --- | --- | --- |
| Species | Buffer size (km) | Deviance  (0-1) | Correlation  (0-1) | Discrimination (AUC)  (0-1) | Kappa (κ)  (-1 to 1) |
| *An. marajoara* (56) | **100** | 0.1750 | 0.7633 | 0.9535 | 0.6342 |
|  | **500** | 0.1575 | 0.7540 | 0.9689 | 0.6636 |
|  | **1000** | 0.1555 | 0.7539 | 0.9745 | 0.7058 |
|  | **1500** | 0.1590 | 0.7592 | 0.9767 | 0.7109 |
| *An. nuneztovari* (171) | **100** | 0.2952 | 0.8333 | 0.9605 | 0.7932 |
|  | **500** | 0.2579 | 0.8389 | 0.9762 | 0.7945 |
|  | **1000** | 0.1736 | 0.9106 | 0.9874 | 0.8795 |
|  | **1500** | 0.2172 | 0.8746 | 0.9814 | 0.8411 |
| *An. pseudopunctipennis* (156) | **100** | 0.2887 | 0.8102 | 0.9656 | 0.7498 |
|  | **500** | 0.2288 | 0.8684 | 0.9765 | 0.8433 |
|  | **1000** | 0.2002 | 0.8738 | 0.9823 | 0.8546 |
|  | **1500** | 0.2191 | 0.8709 | 0.9789 | 0.8472 |
| *An. quadrimaculatus* (379) | **100** | 0.1614 | 0.9471 | 0.9934 | 0.9305 |
|  | **500** | 0.0942 | 0.9702 | 0.9978 | 0.9613 |
|  | **1000** | 0.0794 | 0.9768 | 0.9979 | 0.9744 |
|  | **1500** | 0.0468 | 0.9863 | 0.9992 | 0.9835 |

**Table 4.5**: Evaluation statistics for a range of pseudo-absence:occurrence data ratios and constant values, using 1500 km buffer at 5 x 5 km resolution.

|  |  | Metrics | | | |
| --- | --- | --- | --- | --- | --- |
| Species (no. of presence data) | Pseudo-absence:presence | Deviance  (0-1) | Correlation  (0-1) | Discrimination  (AUC: 0-1) | Kappa (κ)  (-1 to 1) |
| *An. albimanus* (362) | **1:1** | 0.2214 | 0.9419 | 0.9922 | 0.9311 |
|  | **2:1** | 0.1939 | 0.9381 | 0.994 | 0.9191 |
|  | **5:1** | 0.1919 | 0.9026 | 0.9904 | 0.8828 |
|  | **10:1** | 0.118 | 0.894 | 0.9931 | 0.8595 |
|  | **500 points** | 0.2386 | 0.9355 | 0.9882 | 0.9187 |
|  | **1000 points** | 0.2357 | 0.9208 | 0.9884 | 0.9051 |
| *An. albitarsis* (138) | **1:1** | 0.5648 | 0.8277 | 0.9459 | 0.7896 |
|  | **2:1** | 0.3831 | 0.8742 | 0.9704 | 0.8528 |
|  | **5:1** | 0.2322 | 0.8852 | 0.9809 | 0.8548 |
|  | **10:1** | 0.1783 | 0.839 | 0.9811 | 0.7937 |
|  | **500 points** | 0.2596 | 0.8910 | 0.9845 | 0.8612 |
|  | **1000 points** | 0.2201 | 0.8601 | 0.9734 | 0.8154 |
| *An. aquasalis* (57) | **1:1** | 0.4865 | 0.8723 | 0.9677 | 0.8533 |
|  | **2:1** | 0.4737 | 0.8442 | 0.9642 | 0.8266 |
|  | **5:1** | 0.3248 | 0.8232 | 0.9692 | 0.783 |
|  | **10:1** | 0.3144 | 0.6912 | 0.9329 | 0.6142 |
|  | **500 points** | 0.3008 | 0.7569 | 0.9427 | 0.6741 |
|  | **1000 points** | 0.2056 | 0.6975 | 0.9418 | 0.5921 |
| *An. darlingi* (318) | **1:1** | 0.2626 | 0.9257 | 0.9876 | 0.9089 |
|  | **2:1** | 0.2986 | 0.9004 | 0.9839 | 0.8623 |
|  | **5:1** | 0.2382 | 0.8669 | 0.984 | 0.8421 |
|  | **10:1** | 0.1717 | 0.8397 | 0.9845 | 0.7962 |
|  | **500 points** | 0.3112 | 0.8995 | 0.9838 | 0.8651 |
|  | **1000 points** | 0.2591 | 0.8951 | 0.9857 | 0.8694 |
| *An. freeborni* (37) | **1:1** | 0.1712 | 0.9517 | 0.9938 | 0.95 |
|  | **2:1** | 0.1902 | 0.9512 | 0.9938 | 0.9439 |
|  | **5:1** | 0.1203 | 0.931 | 0.995 | 0.9153 |
|  | **10:1** | 0.0649 | 0.957 | 0.9964 | 0.935 |
|  | **500 points** | 0.0554 | 0.9398 | 0.9985 | 0.9183 |
|  | **1000 points** | 0.0476 | 0.9094 | 0.9990 | 0.8892 |

**Table 4.5:** (cont.) Evaluation statistics for a range of pseudo-absence:occurrence data ratios and constant values, using 1500 km buffer at 5 x 5 km resolution.

|  |  | Metrics | | | |
| --- | --- | --- | --- | --- | --- |
| Species (no. of presence data) | Pseudo-absence:presence | Deviance  (0-1) | Correlation  (0-1) | Discrimination (AUC: 0-1) | Kappa (κ)  (-1 to 1) |
| *An. marajoara* (56) | **1:1** | 0.2364 | 0.9274 | 0.9972 | 0.8933 |
|  | **2:1** | 0.4655 | 0.8567 | 0.9685 | 0.8097 |
|  | **5:1** | 0.2991 | 0.8362 | 0.9712 | 0.8162 |
|  | **10:1** | 0.2307 | 0.7788 | 0.9722 | 0.711 |
|  | **500 points** | 0.2216 | 0.8161 | 0.9795 | 0.7458 |
|  | **1000 points** | 0.1590 | 0.7592 | 0.9767 | 0.7109 |
| *An. nuneztovari* (171) | **1:1** | 0.3832 | 0.8832 | 0.98 | 0.8536 |
|  | **2:1** | 0.3405 | 0.8917 | 0.9804 | 0.8527 |
|  | **5:1** | 0.2514 | 0.8689 | 0.9768 | 0.8422 |
|  | **10:1** | 0.1401 | 0.8851 | 0.9866 | 0.8631 |
|  | **500 points** | 0.3593 | 0.8577 | 0.9724 | 0.8292 |
|  | **1000 points** | 0.2172 | 0.8746 | 0.9814 | 0.8411 |
| *An. pseudopunctipennis* (156) | **1:1** | 0.3342 | 0.9049 | 0.9894 | 0.8729 |
|  | **2:1** | 0.3342 | 0.9049 | 0.9894 | 0.8729 |
|  | **5:1** | 0.2056 | 0.9145 | 0.9782 | 0.8927 |
|  | **10:1** | 0.1543 | 0.8678 | 0.9839 | 0.8292 |
|  | **500 points** | 0.3081 | 0.8819 | 0.9765 | 0.8517 |
|  | **1000 points** | 0.2191 | 0.8709 | 0.9789 | 0.8472 |
| *An. quadrimaculatus* (379) | **1:1** | 0.0995 | 0.9806 | 0.9952 | 0.9815 |
|  | **2:1** | 0.0604 | 0.9846 | 0.9989 | 0.9821 |
|  | **5:1** | 0.0716 | 0.9684 | 0.9977 | 0.9587 |
|  | **10:1** | 0.0377 | 0.9722 | 0.999 | 0.966 |
|  | **500 points** | 0.0844 | 0.9760 | 0.9990 | 0.9675 |
|  | **1000 points** | 0.0468 | 0.9863 | 0.9992 | 0.9835 |
